# Reduction-responsive RNAi nanoplatform for enhanced cancer sonoimmunotherapy via dual inhibition of mitophagy and Nrf2 pathways

**DOI:** 10.7150/thno.112649

**Published:** 2025-07-11

**Authors:** Junyue Fang, Rui Xu, Yuan Cao, Zixuan Zhao, Weifan Li, Li Lin, Jingyi Hou, Xiaoding Xu, Phei Er Saw

**Affiliations:** 1 Guangdong Provincial Key Laboratory of Malignant Tumor Epigenetics and Gene Regulation, Medical Research Center, Sun Yat-sen Memorial Hospital, Sun Yat-sen University, Guangzhou 510120, P. R. China.; 2 Guangzhou Key Laboratory of Medical Nanomaterials, Sun Yat-sen Memorial Hospital, Sun Yat-sen University, Guangzhou 510120, P. R. China.; 3 Nanhai Translational Innovation Center of Precision Immunology, Sun Yat-sen Memorial Hospital, Foshan 528200, P. R. China.; 4 Cellular and Molecular Diagnostics Center, Sun Yat-sen Memorial Hospital, Sun Yat-sen University, Guangzhou 510120, P. R. China.; 5 Department of Dermatology, Sun Yat-sen Memorial Hospital, Sun Yat-sen University, Guangzhou 510120, P. R. China.; 6 Department of Orthopedics and Department of Sports Medicine, Sun Yat-sen Memorial Hospital, Sun Yat-sen University, Guangzhou 510120, P. R. China.; 7 Department of General Medicine, Sun Yat-sen Memorial Hospital, Sun Yat-sen University, Guangzhou 510120, P. R. China.

**Keywords:** sonoimmunotherapy, mitophagy inhibition, immune checkpoint blockade, reactive oxygen species, redox-responsive nanoplatform

## Abstract

**Rationale:** Sonodynamic therapy (SDT) has emerged as a promising non-invasive modality with deeper tissue penetration than photodynamic or chemodynamic therapies. However, its therapeutic efficacy remains limited due to inadequate reactive oxygen species (ROS) generation, largely attributed to tumor-intrinsic antioxidant systems and mitophagy. Existing combinations of SDT with immunotherapy are primarily additive and fail to address the mechanistic interplay between ROS suppression and immune evasion.

**Methods:** To overcome these limitations, we developed a redox-responsive RNA interference (RNAi) nanoplatform (NP) for the co-delivery of Nrf2 siRNA, the mitophagy inhibitor 3-Methyladenine (3-MA), and the sonosensitizer purpurin-18 (P-18). This NP enables tumor-specific release in high-glutathione environments and facilitates dual-pathway inhibition upon ultrasound activation.

**Results:** This synergistic platform simultaneously disrupted Nrf2-mediated antioxidant defenses and mitophagy-dependent mitochondrial clearance, resulting in enhanced intracellular ROS accumulation. Elevated ROS levels triggered immunogenic cell death (ICD), promoting dendritic cells maturation and antigen presentation. Concurrently, 3-MA inhibited NF-κB signaling, downregulating PD-L1 expression and mitigating T cell exhaustion. In murine breast cancer models, this dual-action approach elicited robust CD8⁺ T cell responses and significantly suppressed tumor growth and metastasis.

**Conclusions:** This study introduces a mechanistically integrated sonoimmunotherapeutic strategy that concurrently overcomes ROS suppression and immune checkpoint resistance. By orchestrating redox disruption and immune reprogramming, our nanoplatform provides a compelling framework for next-generation SDT-based immunotherapy.

## Introduction

Sonodynamic therapy (SDT) is emerging as a promising non-invasive cancer treatment that leverages ultrasound (US) to activate sonosensitizers, generating reactive oxygen species (ROS) for tumor ablation 1, 2. Compared to photodynamic therapy, which is hindered by limited light penetration, and chemodynamic therapy, which relies on endogenous Fenton reactions, SDT offers superior tissue penetration, spatiotemporal precision, and minimal off-target toxicity 3, 4. Despite these advantages, the clinical translation of SDT remains hampered by several critical limitations, including inefficient ROS production, rapid ROS degradation, limited intracellular delivery, and unwanted ROS consumption 5. These challenges collectively contribute to suboptimal therapeutic outcomes.

A primary obstacle in SDT efficacy lies in the tumor' s intrinsic redox defense mechanisms. The Nrf2-Keap1 pathway serves as a central regulator of antioxidant responses, orchestrating the transcription of detoxifying enzymes such as HO-1, NQO1, and GPX4, which mitigate oxidative stress 6. Concurrently, mitophagy selectively removes damaged mitochondria, a major intracellular source of ROS, further restricting ROS accumulation 7, 8. Together, these pathways form a synergistic network that enables tumor cells to withstand oxidative insults, significantly undermining ROS-dependent therapies like SDT.

To address this limitation, recent research has investigated SDT in combination with immunotherapy, leveraging ROS-induced immunogenic cell death (ICD) to activate antitumor immunity 9. ROS can trigger the release of damage-associated molecular patterns (DAMPs), such as ATP, calreticulin (CRT), and HMGB1, which promote dendritic cells (DCs) maturation and enhance antigen presentation to cytotoxic T lymphocytes (CTLs) 10, 11. However, most SDT-immunotherapy strategies demonstrate only additive effects. These approaches typically focus on single-pathway interventions-such as Nrf2 inhibition or immune checkpoint blockade-without addressing the intricate crosstalk between redox regulation, mitophagy, and immune evasion. Moreover, tumor cells frequently upregulate immune checkpoint proteins such as PD-L1 in response to stress-induced inflammation, establishing a negative feedback loop that suppresses T cell activation and promotes immune escape 12, 13. Consequently, current combination strategies fall short of delivering durable and synergistic therapeutic benefits.

**Scheme 1:**
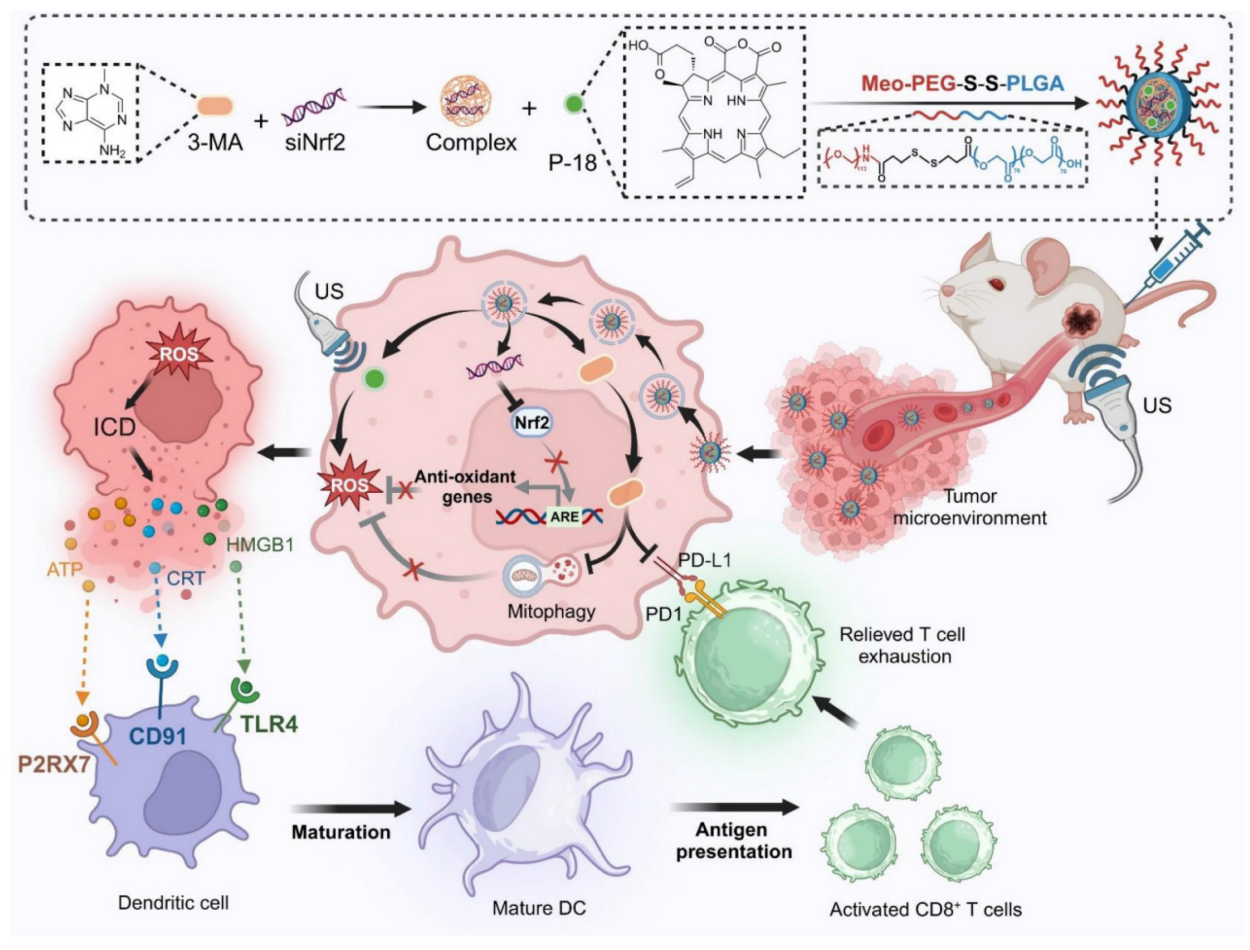
Schematic illustration of the design and therapeutic mechanism of the multifunctional nanoplatform NPs(3-MA/siNrf2/P-18) for enhanced SDT and immune activation. The nanoparticle co-encapsulates 3-MA, siNrf2, and P-18, and is administered intravenously for tumor-targeted delivery. In the reductive tumor microenvironment, intracellular glutathione triggers cleavage of disulfide bonds, facilitating controlled release of the payload. Upon US irradiation, P-18 generates ROS, inducing tumor cell apoptosis. Concurrent Nrf2 silencing attenuates antioxidant defenses, while 3-MA inhibits mitophagy, disrupting mitochondrial clearance and further amplifying intracellular ROS levels. This synergistic ROS accumulation promotes ICD, characterized by the release of DAMPs that enhance DCs maturation and antigen presentation. Additionally, 3-MA downregulates PD-L1 expression via NF-κB pathway inhibition, reversing T cell exhaustion and promoting robust CD8⁺ T cell-mediated cytotoxicity. Together, this strategy enhances SDT efficacy and elicits a potent anti-tumor adaptive immune response, offering a comprehensive approach for improved cancer therapy.

In this study, we present a redox-responsive RNA interference (RNAi) nanoplatform (NP) engineered to co-deliver Nrf2-targeting siRNA (siNrf2), the mitophagy inhibitor 3-Methyladenine (3-MA), and the sonosensitizer purpurin-18 (P-18). The NP features a disulfide-linked architecture that facilitates glutathione (GSH)-triggered release within the tumor microenvironment (TME), ensuring tumor-selective delivery and minimizing systemic toxicity. Upon US activation, this dual-inhibition strategy simultaneously suppresses Nrf2-driven antioxidant responses and mitophagy, resulting in robust intracellular ROS amplification and enhanced ICD. In parallel, 3-MA downregulates PD-L1 expression via NF-κB inhibition, mitigating T cell exhaustion and enhancing CD8⁺ T cell infiltration. By synchronizing redox disruption with immune checkpoint modulation, this integrated approach addresses the core limitations of SDT and immunotherapy, offering a compelling framework for next-generation sonoimmunotherapy. Through targeted modulation of interconnected resistance pathways and TME-responsive delivery, our platform advances the therapeutic landscape toward more effective and mechanistically informed cancer immunotherapy (**Scheme 1**).

## Materials and Methods

### Materials

3-Methyladenine (3-MA), BAY11-7082, Microcystin-LR (MC-LR) and Purpurin 18 (P-18) were purchased from MedChemExpress (MCE) and used without further modification. Dimethyl sulfoxide (DMSO) and N, N'-dimethylformamide (DMF) were acquired from Sigma-Aldrich and used as received. The cationic lipid-like compound alkyl-modified polyamidoamine (PAMAM) dendrimer (G0-C14) and Meo-PEG5k-S-S-PLGA11k copolymer were synthesized according to previously reported procedures 14. The DAB (SA-HRP) TUNEL Cell Apoptosis Detection Kit was purchased from Servicebio^®^. ATP Determination Kit (#A22066), ROS probe (CM-H2DCFDA, #C6827), FIX & PERM™ Cell Permeabilization Kit (#GAS003), MitoTracker® Red CMXRos (#M7512), and LysoTrackerTM Green DND-26 (#L7526) were obtained from Thermo Fisher Scientific. Human CRT (Calreticulin) ELISA Kit (#E-EL-H0627) and Annexin V-FITC/PI Apoptosis Kit (#E-CK-A211) were purchased from Elabscience Biotechnology (Wuhan, China). siRNA targeting human and mouse Nrf2 was acquired from IGE (Guangzhou, China). The siRNA sequences were as follows: siNrf2 (human): 5'-GGC CAG CTG TGA GTG TTT CTT-3' (sense); 5'-AAG AAA CAC TCA CAG CTG GCC-3' (antisense); siNrf2 (mouse): 5'-CAA GGA GCA AUU CAA UGA A-3' (sense); 5'- UUC AUU GAA UUG CUC CUU G-3' (antisense). Cy5-labeled siNrf2 was also obtained from IGE, with the fluorescent dye Cy5 conjugated to the 5'-end of both sense and antisense strands. Dulbecco's Modified Eagle Medium (DMEM), penicillin-streptomycin, trypsin, and fetal bovine serum (FBS) were purchased from Invitrogen. All other reagents and solvents were of analytical grade and used without further purification.

### Antibodies and primers

SQSTM1/p62 rabbit monoclonal antibody (mAb, A19700), LC3A/LC3B rabbit polyclonal antibody (pAb, A5618), and PD-L1/CD274 rabbit pAb (#A1645) were purchased from ABclonal Technology. GAPDH rabbit mAb (GB15004-100) and Cy5-conjugated goat anti-rabbit IgG (H + L) were obtained from Servicebio (Wuhan, China). Horseradish peroxidase (HRP)-conjugated anti-rabbit IgG secondary mAb (#7074) and Ki67 rabbit mAb (#ab92742) were purchased from Cell Signaling Technology (CST) and Abcam, respectively. Additional antibodies obtained from Abcom include: Alexa Fluor® 647-conjugated recombinant anti-calreticulin (CRT) antibody (#ab196159), recombinant anti-Nrf2 antibody (#ab313825), recombinant anti-heme oxygenase 1 antibody (#ab189491), anti-glutathione reductase antibody (#ab124995), anti-NQO1 antibody (#ab80588), anti-superoxide dismutase 3/EC-SOD antibody (#ab80946), and anti-glutathione peroxidase 4 antibody (#ab125066). Flow cytometry antibodies were obtained as follows: from BD Pharmingen -BV510-conjugated rat anti-mouse CD45 (563891), FITC-conjugated rat anti-mouse CD11b (557396), Alexa Fluor 700-conjugated hamster anti-mouse CD3e (557984), Cy5.5-conjugated Rat Anti-Mouse CD8a (551162), BV421-conjugated rat anti-mouse IFN-γ (563376), FITC Rat Anti-Mouse I-A/I-E (2G9) antibody (562009), and PE Hamster Anti-Mouse CD11c (HL3) antibody (557401); from BioLegend-Brilliant Violet 650™anti-mouse CD86c (105036), FITC anti-mouse CD80 Antibody (104706), and APC-conjugated recombinant anti-human/mouse granzyme B (372204). The primers for quantitative reverse transcription polymerase chain reaction (qRT-PCR) are shown in Table 1.

### Preparation and characterization of NP

Reduction-responsive RNAi NP were prepared using a modified nanoprecipitation method as previously described 8, 14, 15. In brief, 3-MA was initially dissolved in DMF, followed by the addition of 1 nM of siPD-L1 (from a 0.1 nM/μL aqueous solution) at varying N/P molar ratios. Subsequently, P-18 in DMF and 200 μL of Meo-PEG-S-S-PLGA copolymer solution (20 mg/mL in DMF) were added to the mixture. The resulting solution was then slowly added dropwise into 5 mL of deionized water under vigorous stirring at 1000 rpm. The formed NP were transferred to an ultrafiltration device (EMD Millipore, MWCO 100 K) and centrifuged to eliminate organic solvents and unbound compounds. After rinsing with deionized water, the final formulation, designated as NPs(3-MA/siNrf2/P-18), was resuspended in deionized water at a siRNA concentration of 1 nM/mL. Control NP, denoted as NPs(G0-C14/siNrf2/P-18), were prepared using the same protocol, substituting 3-MA with the cationic lipid-like compound G0-C14 (5 mg/mL in DMF). The hydrodynamic diameter and zeta potential of all NP were measured by dynamic light scattering (DLS, Malvern, USA), and their morphology was characterized using a transmission electron microscope (TEM, FEI, USA). To evaluate encapsulation efficiency of siRNA, 3-MA, and P-18, Cy5-labeled siNrf2 was encapsulated into NP using the same procedure, yielding NPs(3-MA/Cy5-siNrf2/P-18). A 5 μL aliquot of the NP suspension was diluted 20-fold in DMSO, and the fluorescence intensity of Cy5-labeled siNrf2 was measured. Additionally, UV absorption at 413 nm (for P-18) and 279 nm (for 3-MA) were recorded using a Synergy HT multi-mode microplate reader (BioTek, USA). Encapsulation efficiencies were calculated by comparison with respective standard curves.

### Cell culture

The murine BCa cell line 4T1 and the human BCa cell line MDA-MB-231 were cultured in DMEM supplemented with 10% FBS and 1% penicillin-streptomycin. Cells were maintained at 37 °C in a humidified incubator containing 5% CO_2_. Mouse bone marrow-derived primary cells were cultured under the same conditions using DMEM supplemented with 10% FBS. All animal procedures were approved by the Institutional Review Board (IRB) of Sun Yat-sen Memorial Hospital.

### *In vitro c*argo release

The NPs(3-MA/Cy5-siNrf2/P-18) were dispersed in 1 mL of PBS and transferred into a Float-a-lyzer G2 dialysis device (MWCO 100 kDa, Spectrum). The device was immersed in PBS with or without 10 mM GSH and incubated at 37 ℃. At predetermined time intervals, 5 μL of the NP solution was collected and diluted 20-fold with DMSO. The fluorescence intensity of Cy5-labeled siNrf2, along with UV absorbance at 413 nm for P-18 and 279 nm for 3-MA, was measured using a Synergy HT multi-mode microplate reader. (BioTek, USA). Cumulative release was calculated using the formula: Cumulative release (%) = (Mt / M∞) × 100, where Mt is the amount of Cy5-siNrf2, 3-MA or P-18 released at a time t, and M∞ is the total amount of each cargo initially loaded into the NP.

### *In vitro* Nrf2 silencing and inhibition of downstream genes

4T1 and MDA-MB-231 cells were seeded in 6-well plates at a density of 50,000 cells per well and cultured in 2 mL of DMEM supplemented with 10% FBS for 24 h. Following incubation, NPs(3-MA/siNrf2/P-18) were added to the cells at a final siRNA concentration of 30 nM. After an additional 24 h of incubation, the culture medium was replaced with fresh medium, and cells were further incubated for an additional 48 h. Subsequently, total RNA and protein were extracted from the cells to assess the mRNA and protein expression levels of Nrf2, HO-1, NQO1, GR, SOD, GPX4, and GAPDH, employing qRT-PCR and western blotting, respectively.

### qRT-PCR

Total RNA was isolated from treated cells utilizing Trizol reagent, and 1 μg of total RNA was reverse-transcribed into complementary DNA (cDNA) using the Superscript First-Strand cDNA Synthesis Kit (catalog number 18080-051, Invitrogen, USA), following the manufacturer's protocol. qRT-PCR was carried out employing the SYBR® Premix Ex Taq™ II kit (product code DRR081A, Takara, Japan) on a LightCycler 480 System (Roche, Switzerland).

### Western blot

Protein samples were quantified using a bicinchoninic acid (BCA) protein assay kit (Pierce/Thermo Scientific) according to the manufacturer's protocol. Equal amounts of protein were separated by sodium dodecyl sulfate polyacrylamide gel electrophoresis (SDS-PAGE) and transferred onto a polyvinylidene difluoride (PVDF) membranes. Membranes were blocked in PBS containing 3% bovine serum albumin (BSA) and 0.1% Tween 20 (PBST) for 1 h at room temperature. After blocking, membranes were incubated overnight at 4 ℃ with primary antibody diluted in PBS containing 1% BSA. Following three washes with PBST, membranes were incubated with HRP-conjugated anti-rabbit IgG secondary antibody for 1 h at 4 ℃. After additional washing with PBST, protein bands were visualized using an enhanced chemiluminescence (ECL) detection system. Target proteins included Nrf2, HO-1, NOQ1, GR, SOD, GPX4, GAPDH, PD-L1, p62, LC3, and HMGB1.

### Immunofluorescence (IF)

4T1 and MDA-MB-231 cells were seeded in 6-well plates at a density of 50 000 cells per well and treated with NPs(3-MA/siNrf2/P-18) at a final siRNA concentration of 30 nM as described above. After treatment, cells were fixed with paraformaldehyde (PFA) and permeabilized with 0.2% Triton X-100 in PBS solution for 5 min. Cells were then washed three times with PBS and blocked with PBS containing 3% BSA for 1 h at room temperature. Primary PD-L1 antibody, diluted in PBS solution containing 1% BSA, was added and incubated with the cells for 1 h at 4 ℃. After three PBS washes, cells were incubated with Alexa Fluro 647-conjugated secondary antibody for 1 h at 4 ℃. After another set of PBS washes, nuclei were stained with Hoechst 33342. Fluorescence imaging was performed using a ZEISS 800 confocal laser scanning microscope (CLSM).

### *In vitro* inhibition of mitophagy

4T1 and MDA-MB-231 cells were seeded into 6-well plates at a density of 50 000 cells per well and treated with NPs(3-MA/siNrf2/P-18) at a final siRNA concentration of 30 nM as previously described. After treatment, cells were stained with MitoTracker® Red CMXRos and LysoTracker™ Green DND-26 to evaluate the co-localization of lysosomes and mitochondria using CLSM. Following imaging, cells were harvested by trypsinization and total protein was extracted for western blot analysis of p62 and LC3 I/II expression.

### Detection of intracellular ROS levels

4T1 and MDA-MB-231 cells were seeded into 6-well plates at a density of 50000 cells per well and treated with NPs(3-MA/siNrf2/P-18) under US irradiation, using a final siRNA concentration of 30 nM, as previously outlined. After treatment, cells were incubated with 5 μM CM-H2DCFDA, a ROS-sensitive fluorescent probe, for 15 min at 37 ℃. Intracellular ROS levels were visualized using CLSM. Following imaging, cells were harvested and analyzed by flow cytometry utilizing a BD FACSAria™ III flow cytometry to quantitatively assess ROS accumulation.

### Detection of ATP and CRT release

4T1 and MDA-MB-231 cells were seeded in 6-well plates at a density of 50 000 cells per well and treated with NPs(3-MA/siNrf2/P-18) under US irradiation at a final siRNA concentration of 30 nM, accordingly to the protocol described above. After treatment, the cell culture supernatants were collected for quantification of ATP and CRT levels. ATP concentration was measured using the ATP Determination Kit, while CRT levels were assessed using both the human CRT ELISA Kit (#E-EL-H0627) and Recombinant Alexa Fluor® 647 anti-CRT antibody, according to the manufacturer's protocol.

### *In vitro* proliferation and colony formation

4T1 and MDA-MB-231 cells were seeded in 6-well plates at a density of 20,000 cells per well and cultured in 2 mL of DMEM supplemented with 10% FBS for 24 h. Cells were then treated with NPs(G0-C14/siNrf2/P-18), NPs(3-MA/siCTL/P-18), or NPs(3-MA/siNrf2/P-18) at a siRNA concentration of 30 nM, with or without US irradiation (3 min, 1 W/cm², 3 MHz, and 50% duty cycle). After 24 h of incubation, cells were rinsed with PBS, and cell viability was assessed using the Alamar Blue assay according to the manufacturer's protocol. After the viability measurement, the Alamar Blue reagent was removed and cells were cultured in fresh medium. For the colony formation assay, MDA-MB-231 and 4T1 cells were seeded in 6-well plates at a density of 2,000 cells per well. The cells were treated with the same nanoparticle formulations and conditions as described above. After seven days of incubation in complete medium, colonies were fixed and stained with crystal violet. Colony formation was observed using an MVX10 Macro View Dissecting Scope equipped with an Olympus DP80 camera.

### Apoptosis analysis

MDA-MB-231 and 4T1 cells were seeded into 6-well plates at a density of 50,000 per well and cultured in 2 mL of DMEM supplemented with 10% FBS for 24 h. Cells were then treated with NPs(G0-C14/siNrf2/P-18), NPs(3-MA/siCTL/P-18), or NPs(3-MA/siNrf2/P-18) at a final siRNA concentration of 30 nM with or without US irradiation, as described previously. After 24 h, cells were washed with PBS, incubated in fresh medium for an additional 24 h, harvested, and stained using the Annexin V-FITC/PI Apoptosis Detection Kit (AK12637, Elabscience). Apoptosis was quantified by flow cytometry using a CytoFlex LX Flow Cytometry Analyzer (Beckman Coulter).

### Animals

Healthy female BALB/c mice (4-5 weeks old) were purchased from Sun Yat-sen University Experimental Animal Center (Guangzhou, China). All animal experiments were conducted in accordance with protocols approved by the Institutional Animal Care and Use Committee of Sun Yat-sen Memorial Hospital (#AEP20240215).

### Pharmacokinetics

Healthy female BALB/c mice were randomly divided into three groups (n = 3) and administered an intravenous injection of one of the following formulations: (i) naked Cy5-labled siNrf2, or (ii) NPs(3-MA/Cy5-labled siNrf2/P-18) at a dose of 5 mg/kg P-18 and/or 6 mg/kg 3-MA and/or 1 nM siNrf2 per mouse. At predetermined time intervals post-injection, 20 μL of blood was collected via the orbital vein. The fluorescence intensity of Cy5-labeled siNrf2 in the blood samples was measured using fluorescence spectroscopy to assess circulation kinetics.

### Orthotopic and lung metastatic tumor model

To establish the 4T1 orthotopic tumor model, 200 μL of a 4T1 cell suspension-comprising a 1:1 volume mixture of DMEM and Matrigel with a cell concentration of 1×10^7^ cells/mL-was subcutaneously injected into the second pair of mammary fat pads of healthy female BALB/c mice. Once tumors reached approximately 100 mm^3^ in volume, these tumor-bearing mice were enrolled in subsequent *in vivo* experiments. For the lung metastasis model, 2×10^5^ luciferase-expressing 4T1 cells suspended in 100 μL of PBS were administered intravenously into healthy mice. Tumor progression was tracked by bioluminescence imaging using an IVIS Lumina III system (PerkinElmer, USA). Prior to imaging, D-luciferin was administered via intraperitoneal injection at a dose of 150 mg/kg. Average radiance values at tumor sites were used to quantify tumor burden.

### Biodistribution

4T1 orthotopic tumor-bearing mice were randomly divided into two groups (n = 3) and administered intravenous injections of either: (i) unencapsulated Cy5-labeled siNrf2, or (ii) NPs(3-MA/Cy5-labeled siNrf2/P-18) at a dosage of 5 mg/kg P-18 and/or 6 mg/kg 3-MA and/or 1 nM siNrf2 per mouse. At 24 h post-injection, tumors and major organs were harvested and imaged using the IVIS Lumina III system (PerkinElmer, USA). The accumulation of Cy5-labeled siNrf2 in tumor and organ tissues was quantified based on fluorescence intensity using Image J software.

### *In vivo* PD-L1 downregulation and Nrf2 silencing

4T1 orthotopic tumor-bearing mice were randomly divided into six groups (n = 5) and treated with daily intravenous injections of the following formulations: (i) PBS, (ii): NPs(3-MA/siCTL/P-18), (iii) NPs(G0-C14/siNrf2/P-18), (iv) NPs(3-MA/siCTL/P-18) + US, (v) NPs(G0-C14/siNrf2/P-18) + US, and (vi) NPs(3-MA/siNrf2/P-18) + US. Each injection was administered at a dose of 5 mg/kg P-18 and/or 6 mg/kg 3-MA and/or 1 nM siNrf2 per mouse. After three consecutive injections, mice were sacrificed 24 h following the final dose. Tumors were excised, and total protein was extracted for western blot analysis of PD-L1 and Nrf2 expression. In parallel, tumor tissues were homogenized into single-cell suspensions for flow cytometry analysis to evaluate DCs maturation, CD8^+^ T cell infiltration, and granzyme B and IFN-γ production by CD8^+^ T cells, according to the manufacturer's protocol.

### Inhibition of orthotopic tumor growth

4T1 orthotopic tumor-bearing mice were randomly divided into six groups (n = 5) and treated with four consecutive intravenous injections of the following formulations: (i) PBS, (ii) NPs(3-MA/siCTL/P-18), (iii) NPs(G0-C14/siNrf2/P-18), (iv) NPs(3-MA/siCTL/P-18) + US, (v) NPs(G0-C14/siNrf2/P-18) + US, and (vi) NPs(3-MA/siNrf2/P-18) + US. Injections were administered once every two days at a dose of 5 mg/kg P-18 and/or 6 mg/kg 3-MA and/or 1 nM siNrf2 per mouse. Tumor growth was monitored every two days by measuring the shortest (W) and longest (L) diameters with a caliper. Tumor volume was calculated using the formula: V = W^2^×L/2. At the experimental endpoint, tumors were collected and sectioned for TUNEL and Ki67 immunohistochemical staining according to the manufacturer's protocol.

### Inhibition of lung metastatic tumor growth

4T1 lung metastasis tumor-bearing mice were randomly assigned into six groups (n = 5) and treated with four consecutive intravenous injections of the following: (i) PBS, (ii) NPs(3-MA/siCTL/P-18), (iii) NPs(G0-C14/siNrf2/P-18), (iv) NPs(3-MA/siCTL/P-18) + US, (v) NPs(G0-C14/siNrf2/P-18) + US, and (vi) NPs(3-MA/siNrf2/P-18) + US. The injections were administered once every two days at a dose of 5 mg/kg P-18 and/or 6 mg/kg 3-MA and/or 1 nM siNrf2 per mouse. Lung tumor progression was monitored on days 0, 7, and 14 using a bioluminescence imaging system, following the procedures previously described. At the conclusion of the experiment, tumors were excised and sectioned for TUNEL and Ki67 staining, in accordance with the manufacturer's instructions.

### Immunohistochemistry (IHC)

IHC staining was conducted on formalin-fixed, paraffin-embedded tumor sections. In summary, tumor slides were initially heated to 60 °C for 1 h, followed by deparaffinization using xylene (three washed, 5 min each) and rehydration through a graded ethanol alcohol series. Antigen retrieval was performed using DAKO Target Retrieval Solution at 95-99 ℃ for 40 min, followed by washing in distilled water. Endogenous peroxidase activity was blocked using DAKO peroxidase blocking buffer for 5 min. After washing, slides were incubated with the appropriate primary antibody diluted in DAKO antibody diluent for 1 h at room temperature. Slides were then washed and incubated with a peroxidase-conjugated polymer for 30 min. Following a final wash, staining was developed using DAB^+^ substrate-chromogen solution and counterstained with hematoxylin. Stained slides were mounted and imaged using an MVX10 MacroView Dissecting Scope equipped with an Olympus DP80 camera.

### Blood and histological analysis

Healthy female BALB/c mice were randomly divided into six groups (n = 3) and treated with intravenous injections of the following formulations: (i) PBS, (ii) NPs(3-MA/siCTL/P-18), (iii) NPs(G0-C14/siNrf2/P-18), (iv) NPs(3-MA/siCTL/P-18) + US, (v) NPs(G0-C14/siNrf2/P-18) + US, and (vi) NPs(3-MA/siNrf2/P-18) + US. Following three consecutive daily injections, blood samples were collected 24 h after the final dose, and serum was isolated for analysis of standard hematological and biochemical parameters. Major organs, including the heart, liver, spleen, lung, and kidneys, were harvested and processed for histological examination.

### Statistical analysis

All quantitative data are presented as mean ± standard deviation (SD) from at least three independent experiments. Graphpad Prism software (version 8.0) was used for data visualization, statistical analysis, and figure generation. Specific sample sizes used for each experiment are detailed in the corresponding figure legends. Statistical comparisons between two groups were performed using two-tailed Student's *t*-test, while comparisons among multiple groups were conducted using one-way ANOVA. A P-value < 0.05 was considered statistically significant.

## Results and Discussion

### Preparation and characterizations of NPs(3-MA/siNrf2/P-18)

**Figure 1:**
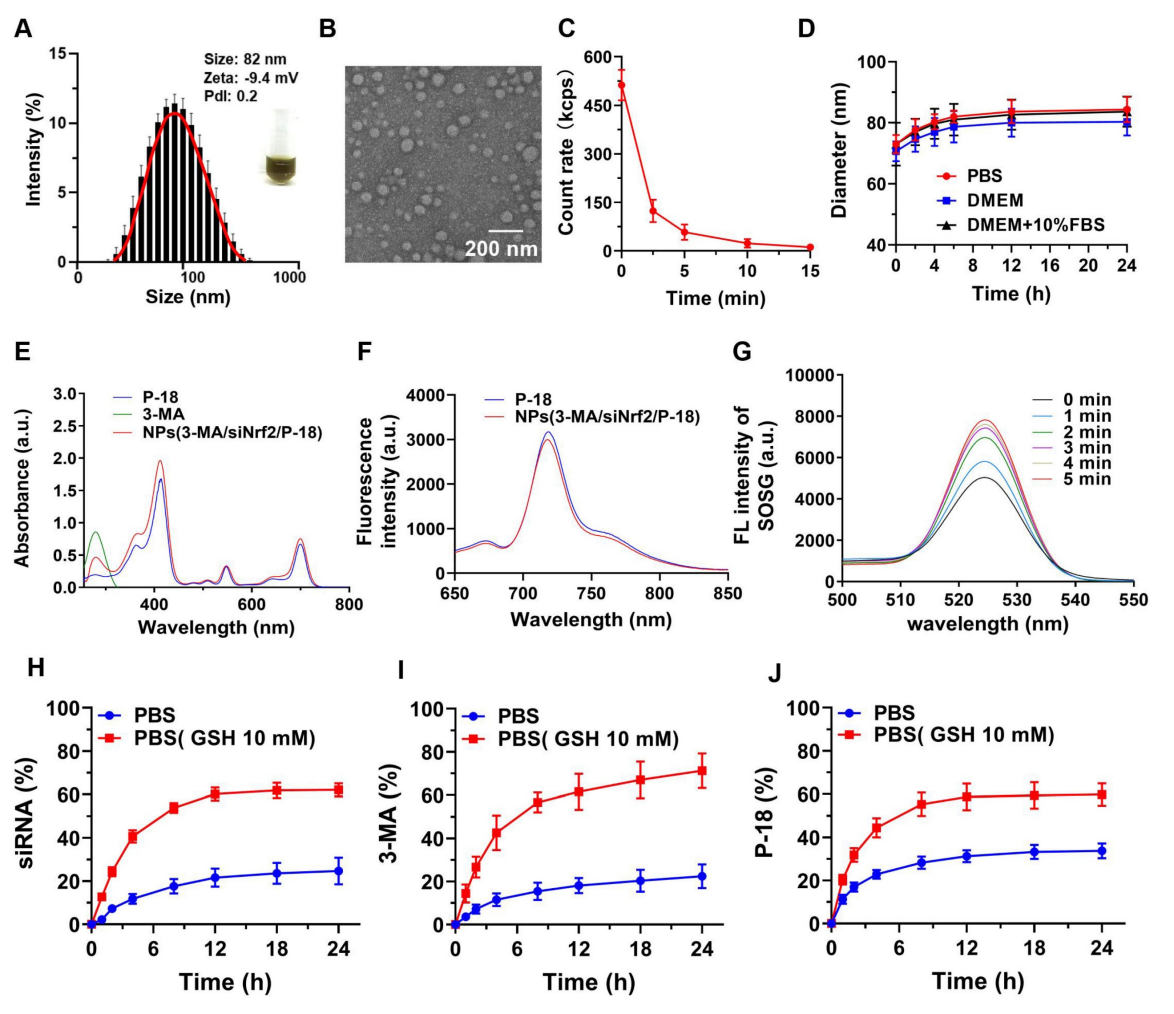
Characterization and stability of NPs(3-MA/siNrf2/P-18). (A) DLS analysis showing the hydrodynamic size distribution of NPs(3-MA/siNrf2/P-18). (B) TEM image illustrating the morphology and uniform size of the nanoparticles (scale bar: 200 nm). (C) Quantitative analysis of nanoparticle degradation over time in the presence of 10 mM glutathione (GSH), simulating reductive tumor microenvironment conditions. (D) Assessment of nanoparticle size stability following incubation in PBS, DMEM, and DMEM supplemented with 10% FBS over various time points. (E) UV-Vis absorption spectra confirming successful encapsulation of P-18 and characterization of optical properties. (F) Fluorescence emission spectra of free P-18 versus P-18-loaded nanoparticles in DMSO, indicating retained photophysical properties post-encapsulation. (G) Singlet oxygen generation, measured via SOSG fluorescence, from NPs(3-MA/siNrf2/P-18) subjected to ultrasound (US) irradiation for varying durations. (H-J) Cumulative release profiles of siRNA, 3-MA, and P-18 from the nanoplatform over 24 hours in PBS and GSH-containing PBS (10 mM), demonstrating redox-responsive drug release behavior.

To facilitate clinical translation, a simplified and efficient method was employed for the synthesis of a reduction-responsive RNAi NP. The NP were prepared using a modified nanoprecipitation method 16-18, in which the amphiphilic copolymer Meo-PEG-S-S-PLGA and the sonosensitizer P-18 were dissolved in dimethyl formamide (DMF) and subsequently mixed with aqueous solutions of siNrf2 and 3-MA. The mixture was then added dropwise into deionized water under vigorous stirring. In aqueous conditions, the Meo-PEG-S-S-PLGA polymer self-assembled into spherical NP featuring a hydrophobic PLGA inner core and hydrophilic PEG outer shell 17. Within this formulation, 3-MA formed electrostatic complexes with siNrf2, enabling co-encapsulation within the PLGA core alongside P-18 (Scheme 1). By adjusting the feed ratio of 3-MA to siNrf2 (**Figure S1, Supporting Information**), an optimal nitrogen-to-phosphate (N/P) molar ratio of 95:1 was selected, yielding well-defined spherical NPs(3-MA/siNrf2/P-18) with an average diameter of ~82 nm (**Figure 1A**-**1B; Figure R, Supporting Information**). These NP demonstrated high encapsulation efficiencies: ~76% for siNrf2, ~93% for 3-MA, and ~62% for P-18. UV absorbance spectra confirmed successful loading, with characteristic peaks at 413 nm, 548 nm, and 700 nm for P-18, and 279 nm for 3-MA (**Figure 1E**). Fluorescence (FL) analysis revealed an emission peak at 720 nm for the NP, corresponding to the free P-18 spectrum, further confirming its successful incorporation (**Figure 1F**). As a control, NPs(G0-C14/siNrf2/P-18) were synthesized by replacing 3-MA with the amphiphilic cationic compound G0-C14, developed in a previous study (**Figure S2, Supporting Information**) 19-21. The redox-responsiveness of the NP was validated by its structural disassembly in the presence of 10 mM glutathione (GSH), which mimics the TME (**Figure 1C**), while stability was retained under normal physiological conditions (**Figure 1D**). This disassembly triggered the rapid release of siNrf2 (**Figure 1H**), 3-MA (**Figure 1I**), and P-18 (**Figure 1J**). The NPs(3-MA/siNrf2/P-18) exhibited robust colloidal stability in PBS, DMEM, and 10% FBS over 24 h, with minimal variation in particle size, attributed to the PEGylated surface providing steric hindrance that mitigates protein adsorption and aggregation-a well-documented strategy for prolonged circulation 22. The disulfide linker in Meo-PEG-S-S-PLGA enabled selective drug release under TME conditions, where elevated GSH levels (~10 mM) trigger rapid NP disassembly. This dual-functionality design-PEG-mediated circulation stability and TME-responsive release-minimizes off-target leakages while maximizing tumor-specific drug delivery, consistent with previous reports 17. Plasma protein interaction studies showed only slight increases in NP size after 24 h of incubation in mouse plasma, indicating strong anti-fouling properties (**Figure S3, Supporting Information**). The low protein binding was attributed to the hydrophilic PEG corona of Meo-PEG-S-S-PLGA, which forms a “stealth” protective layer through steric hindrance and hydrogen bonding, thereby limiting opsonin adsorption (e.g., immunoglobulins and complement proteins) and reducing recognition by the reticuloendothelial system 22. These results collectively support the suitability of the NP as a stable and effective delivery vehicle for cancer therapy.

To evaluate the sonodynamic performance of the P-18-loaded NP, singlet oxygen generation was assessed under US irradiation (3 min, 1 W/cm^2^, 3 MHz, and 50% duty cycle). The singlet oxygen sensor green (SOSG) probe was employed to monitor singlet oxygen levels. Upon US exposure, a sharp increase in SOSG absorbance was observed over time, indicating progressive generation of singlet oxygen by NPs(3-MA/siNrf2/P-18) (**Figure 1G**). These findings confirmed that the NP possesses excellent sonodynamic properties and can effectively induce high levels of ROS under US stimulation.

### *In vitro* Nrf2 silencing and cellular functional assessment of reduction-responsive RNAi NP

**Figure 2:**
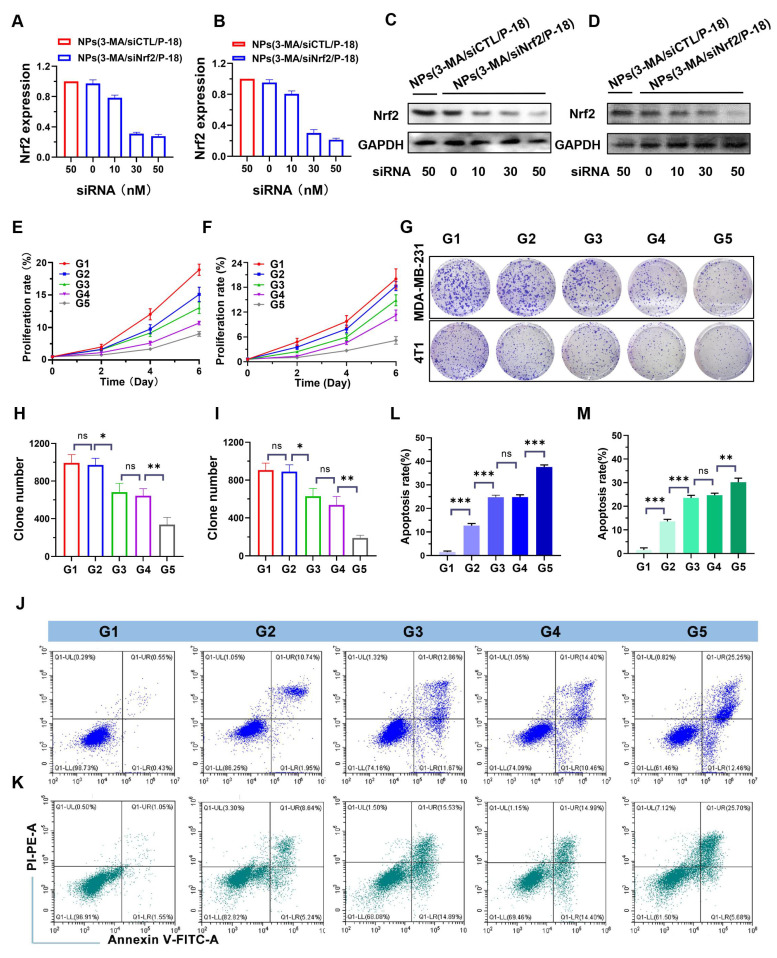
*In vitro* evaluation of Nrf2 silencing, proliferation inhibition, and apoptosis induction by NPs(3-MA/siNrf2/P-18). (A, B) Quantitative real-time PCR (qRT-PCR) analysis of relative Nrf2 mRNA expression in MDA-MB-231 and 4T1 cells, respectively, following treatment with NPs(3-MA/siNrf2/P-18). (C, D) Western blot analysis of Nrf2 protein levels in MDA-MB-231 and 4T1 cells after the indicated treatments. (E, F) Cell proliferation assays showing growth inhibition in MDA-MB-231 and 4T1 cells treated with NPs(3-MA/siNrf2/P-18) with or without ultrasound (US) irradiation. (G) Representative colony formation assay images and (H, I) Quantitative analysis of colony numbers in MDA-MB-231 and 4T1 cells, respectively, following treatment with NPs(3-MA/siNrf2/P-18) under US irradiation. (J-M) Flow cytometry analysis of apoptosis rates in MDA-MB-231 and 4T1 cells after treatment with NPs(3-MA/siNrf2/P-18) with US activation. G1: Blank; G2: NPs(3-MA/siNrf2/P-18); G3: NPs(3-MA/siCTL/P-18) + US; G4: NPs(G0-C14/siNrf2/P-18) + US; G5: NPS(3-MA/siNrf2/P-18) + US. Data are presented as mean ± SD (n = 3). Error bars represent standard deviation. Statistical analysis was performed using one-way ANOVA for multiple comparisons. Significance is indicated as *P < 0.05, **P < 0.01 and ***P < 0.001, ns, no significance.

Following the successful development of the reduction-responsive RNAi NP, its ability to silence Nrf2 expression was evaluated. NPs(3-MA/siNrf2/P-18) were incubated with human-derived BCa cells (MDA-MB-231) and mouse-derived BCa cells (4T1) to assess Nrf2 knockdown efficiency. Nrf2 is often overexpressed in cancer cells and has been associated with the promotion of angiogenesis, drug resistance, cancer stem cell formation, and metastasis 23. Aberrant expression of Nrf2 contributes to decreased therapeutic efficacy and confers cytoprotective advantages to tumor cells. Therefore, Nrf2 knockdown is considered a promising strategy to disrupt these cancer-promoting pathways. Quantitative results indicated that treatment with NPs(3-MA/siNrf2/P-18) led to a dose-dependent decrease in Nrf2 mRNA levels in both MDA-MB-231 (**Figure 2A**) and 4T1 cells (**Figure 2B**). A corresponding reduction in Nrf2 protein expression was also observed in MDA-MB-231 (**Figure 2C**) and 4T1 cells (**Figure 2D**) following NPs(3-MA/siNrf2/P-18) treatment, confirming efficient RNA interference after cellular uptake of the NP. In addition to Nrf2 silencing, functional studies demonstrated that these nanoparticles significantly inhibited cell proliferation and induced apoptosis under US irradiation. As shown in **Figure 2E** and **Figure 2F**, treatment with NPs(3-MA/siNrf2/P-18) markedly reduced proliferation in MDA-MB-231 (**Figure 2E**) and 4T1 cells (**Figure 2F**), by approximately fivefold relative to controls. The results of the colony formation assay (**Figure 2G**) further supported these findings, showing that US-activated NPs(3-MA/siNrf2/P-18) treatment significantly suppressed long-term growth potential in MDA-MB-231 (**Figure 2H**) and 4T1 cells (**Figure 2I**). Furthermore, flow cytometry analysis indicated a pronounced increase in apoptosis upon treatment with NPs(3-MA/siNrf2/P-18) in both cell lines, attributed to combined mitophagy inhibition and Nrf2 silencing. This effect was enhanced under US-induced sonodynamic therapy conditions, as evidenced by apoptosis rates in MDA-MB-231 (**Figure 2J** and **Figure 2L**) and 4T1 cells (**Figure 2K** and **Figure 2M**). Collectively, these findings suggest that the reduction-responsive RNAi NP effectively silences Nrf2 expression, suppresses cellular proliferation, and enhances apoptosis in BCa cells through the synergistic effects of RNA interference, mitophagy inhibition by 3-MA, and sonodynamic ROS induction via P-18.

### Enhancing sonodynamic therapy and ICD through dual inhibition of mitophagy and Nrf2 pathways

Mitochondrial autophagy (mitophagy) serves as an important quality control mechanism that eliminates damaged mitochondria and excess ROS, thereby preserving cellular homeostasis 7. Beyond this protective role, mitophagy helps limit the deleterious effects of ROS accumulation. Inhibition of mitophagy has been shown to enhance ROS levels within cells 24, 25. Therefore, the potential of the reduction-responsive RNAi NP to simultaneously disrupt mitophagy and the Nrf2-associated antioxidant system in BCa cells was evaluated to determine its capacity to potentiate SDT-mediated ROS accumulation. To explore the impact of Nrf2 silencing on mitochondrial function, JC-1 staining and MitoSOX Red assays were performed. JC-1 staining revealed a significant reduction in the red/green fluorescence ratio in both MDA-MB-231 and 4T1 cells following Nrf2 knockdown (**Figure S4, Supporting Information**), indicating mitochondrial membrane potential (ΔΨm) collapse. This effect was further exacerbated under US irradiation, confirming that Nrf2 deficiency sensitizes mitochondria to SDT-induced damage. MitoSOX Red staining demonstrated that mtROS levels increased substantially upon Nrf2 silencing, with combined SDT treatment inducing a 2.8-fold increase in 4T1 cells and a 3.4-fold increase in MDA-MB-231 cells (**Figure S5, Supporting Information**), thereby overwhelming cellular antioxidant defenses. These findings demonstrate that Nrf2 silencing disrupts mitochondrial redox balance through two interrelated mechanisms: (i) antioxidant Depletion-loss of key enzymes such as SOD2 and GPX1 impairs mtROS detoxification 26; and (ii) metabolic destabilization-mtROS overload destabilizes ETC complexes, resulting in exacerbated electron leakage and propagation of oxidative stress, an effect further intensified by mitophagy inhibition 27. This dual inhibition strategy establishes a self-amplifying cycle of mitochondrial damage, underscoring the necessity of targeting both pathways to achieve irreversible mitochondrial dysfunction and optimize SDT efficacy.

**Figure 3:**
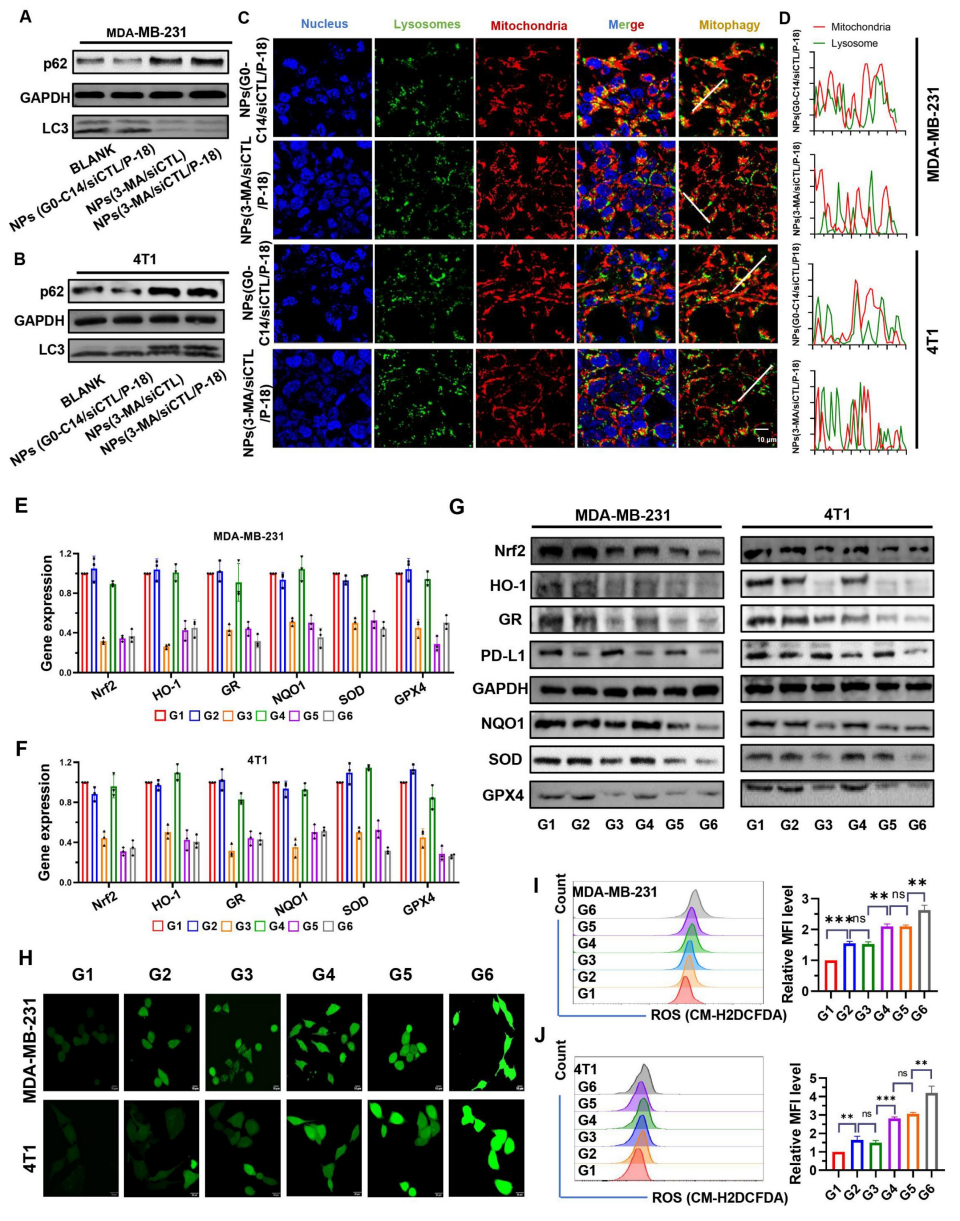
Evaluation of autophagy inhibition, antioxidant gene regulation, PD-L1 expression, and ROS accumulation following treatment with NPs(3-MA/siNrf2/P-18). (A, B) Western blot analysis of autophagy-related proteins p62 and LC3 in MDA-MB-231 and 4T1 cells after treatment with NPs(3-MA/siNrf2/P-18), indicating mitophagy inhibition. (C, D) Confocal microscopy images showing colocalization of mitochondria and lysosomes in MDA-MB-231 and 4T1 cells, respectively, demonstrating altered mitophagy dynamics following treatment. (E, F) Quantitative RT-PCR analysis of antioxidant-related gene expression (Nrf2, HO-1, GR, NQO1, SOD, and GPX4) in MDA-MB-231 and 4T1 cells after nanoparticle treatment. (G) Western blot analysis of PD-L1 and antioxidant protein levels (Nrf2, HO-1, GR, NQO1, SOD, and GPX4) in both cell lines post-treatment, indicating immunomodulatory and redox-disruptive effects. (H) Confocal fluorescence imaging of intracellular ROS generation in MDA-MB-231 and 4T1 cells following US-activated treatment with NPs(3-MA/siNrf2/P-18). (I, J) Flow cytometry analysis and quantification of ROS levels in MDA-MB-231 and 4T1 cells, respectively, after various treatments with or without ultrasound irradiation. Treatment groups: G1: PBS; G2: NPs(3-MA/siCTL/P-18); G3: NPs(G0-C14/siNrf2/P-18); G4: NPs(3-MA/siCTL/P-18) + US; G5: NPs(G0-C14/siNrf2/P-18) + US; G6: NPs(3-MA/siNrf2/P-18) + US. Data are presented as mean ± SD (n = 3). Error bars represent standard deviation. Statistical analysis was performed using one-way ANOVA for multiple comparisons. Significance levels are indicated as *P < 0.05, **P < 0.01 and ***P < 0.001, ns, no significance.

**Figure 4:**
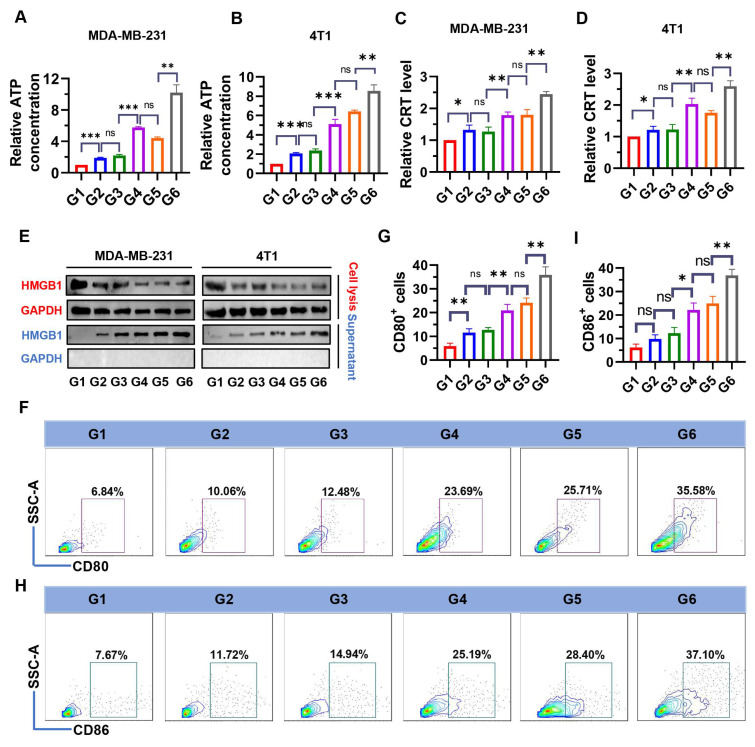
Evaluation of ICD markers and DCs activation following NPs(3-MA/siNrf2/P-18) treatment under ultrasound (US) irradiation. (A, B) Quantification of extracellular ATP levels in the supernatant of MDA-MB-231 and 4T1 cells, respectively, determined by ELISA after treatment with NPs(3-MA/siNrf2/P-18) with US activation. (C, D) Flow cytometry analysis of CRT surface exposure on MDA-MB-231 and 4T1 cells, respectively, indicating ICD induction. (E) HMGB1 protein levels in cell supernatants, measured by ELISA, from MDA-MB-231 and 4T1 cells post-treatment under US irradiation. (F, G) Percentage of CD80⁺ DCs and corresponding quantitative analysis following co-culture with pretreated 4T1 tumor cells, indicating enhanced DC maturation. (H, I) Percentage of CD80⁺ DCs and corresponding statistical analysis following co-culture under the same conditions, further confirming DC activation. Treatment groups: G1: PBS; G2: NPs(3-MA/siCTL/P-18); G3: NPs(G0-C14/siNrf2/P-18); G4: NPs(3-MA/siCTL/P-18) + US; G5: NPs(G0-C14/siNrf2/P-18) + US; G6: NPs(3-MA/siNrf2/P-18) + US. Data are presented as mean ± SD (n = 3). Error bars represent standard deviation. Statistical analysis was performed using one-way ANOVA for multiple comparisons. Significance levels are indicated as *P < 0.05, **P < 0.01 and ***P < 0.001, ns, no significance.

p62 is a well-established substrate for autophagy, and its expression level is inversely correlated with autophagic activity 28. During autophagy, cytoplasmic LC3-I undergoes enzymatic processing and is subsequently conjugated to phosphatidylethanolamine (PE), forming the membrane-associated LC3-II, which is recruited to autophagosomal membranes 29. Therefore, the LC3-II/I ratio serves as a widely used indicator of autophagic flux. Treatment with 3-MA loaded NP resulted in increased p62 accumulation and a decreased LC3-II/LC3-I ratio in both MDA-MB-231 (**Figure 3A**) and 4T1 cells (**Figure 3B**), indicating reduced autophagy levels. Moreover, compared to NPs(G0-C14/siCTL/P-18), treatment with NPs(3-MA/siCTL/P-18) inhibited the colocalization of mitochondria and lysosomes in both cell lines (**Figure 3C-3D**), confirming successful mitophagy inhibition by the 3-MA loaded formulation. 3-MA, a PI3K inhibitor, is widely used to inhibit autophagy by targeting class I PI3K 30. These results confirm that the synthesized NP effectively replicates the autophagy-inhibitory function of the free drug 3-MA. Moreover, the expression of Nrf2-regulated downstream antioxidant genes-including heme oxygenase-1 (HO-1) 31, 32, glutathione reductase (GR) 33, NAD(P)H: quinone oxidoreductase (NQO1) 34, 35, superoxide dismutase (SOD), and glutathione peroxidase 4 (GPX4) 36, 37, was significantly downregulated at both the mRNA (**Figure 3E-3F**) and protein levels (**Figure 3G**) following Nrf2 silencing. In addition, PD-L1 expression was also reduced, suggesting that Nrf2 may contribute to the regulation of immune checkpoint molecules. This is consistent with previous reports indicating 3-MA can downregulate PD-L1 expression via an NF-κB-dependent pathway 38, 39. To further confirm the mechanism, BAY11-7082, a NF-κB inhibitor, was used to mimic the effect of 3-MA and 3-MA-containing NP on the NF-κB/PD-L1 signaling axis and p-p65 nuclear localization. These inhibitory effects were reversed by MC-LR, a phosphatase inhibitor that indirectly promotes p65 phosphorylation by suppressing dephosphorylation, indicating that both 3-MA and 3-MA-loaded NP downregulate PD-L1 expression via the NF-κB pathway (**Figure S6, Supporting Information**). To exclude the possibility that G0-C14 itself influences mitophagy or PD-L1 expression, MDA-MB-231 and 4T1 cells were treated with various concentrations of G0-C14. Western blot analysis showed no significant changes in LC3, p62, or PD-L1 levels (**Figure S7, Supporting Information**), confirming that G0-C14 functions solely as a cationic carrier to enhance siRNA delivery, without directly affecting autophagic flux or immune checkpoint regulation.

Following confirmation of Nrf2 and mitophagy inhibition in BCa cells, intracellular ROS levels were evaluated via confocal fluorescence imaging (**Figure 3H**) and flow cytometry (**Figure 3I-3J**). Both methods demonstrated significantly increased ROS accumulation in MDA-MB-231 and 4T1 cells after treatment with NPs(siNrf2/3-MA/P-18) under US irradiation. In summary, the reduction-responsive RNAi NP facilitates cascade amplification and accumulation of ROS within tumor cells by simultaneously silencing Nrf2 silencing and inhibiting mitochondrial autophagy.

Increased ROS are an important cause of ICD induction 40. To examine whether NPs(3-MA/siNrf2/P-18) could trigger intensive ICD, the release of DAMPs, including ATP, CRT, and HMGB1 were evaluated in dying BCa cells. As shown in **Figure 4**, treatment with NPs(3-MA/siNrf2/P-18) under US irradiation significantly promoted ATP released from MDA-MB-231 (**Figure 4A**) and 4T1 cells (**Figure 4B**), as well as CRT exposure in MDA-MB-231 (**Figure 4C**) and 4T1 cells (**Figure 4D**). In addition, HMGB1 protein levels in the culture supernatant were significantly increased following NPs(3-MA/siNrf2/P-18) treatment with US irradiation (**Figure 4E**), indicating enhanced passive cell death. These results demonstrate that SDT induced by NPs(3-MA/siNrf2/P-18) effectively promotes apoptosis and ICD, leading to substantial release of DAMPs from BCa cells. DAMPs released during ICD are recognized by pattern recognition receptors (PRRs) on the surface of DCs, initiating a series of cytological responses that ultimately activate both innate and adaptive immune responses 14. To determine whether NPs(3-MA/siNrf2/P-18)-induced ICD could successfully activate DCs *in vitro*, a co-culture system was established using mouse-derived DCs and 4T1 cells in a Boyden chamber device (**Figure S8, Supporting Information**). Following treatment with NPs(3-MA/siNrf2/P-18) and US irradiation, a significant increase in the percentage of CD80^+^ and CD86^+^ DCs was observed-by approximately fivefold-compared to controls (**Figure 4F-4I**), indicating robust DCs activation. To rule out the possibility that US alone contributed to ICD induction, an additional control group with US-only treatment was included. 4T1 and MDA-MB-231 cells were exposed to the same US conditions described in the study, and ICD markers were assessed. The results confirmed that US treatment alone did not induce significant changes in ICD indicators (**Figure S9, Supporting Information**).

### Antitumor effect of NPs(3-MA/siNrf2/P-18)* in vivo*

**Figure 5:**
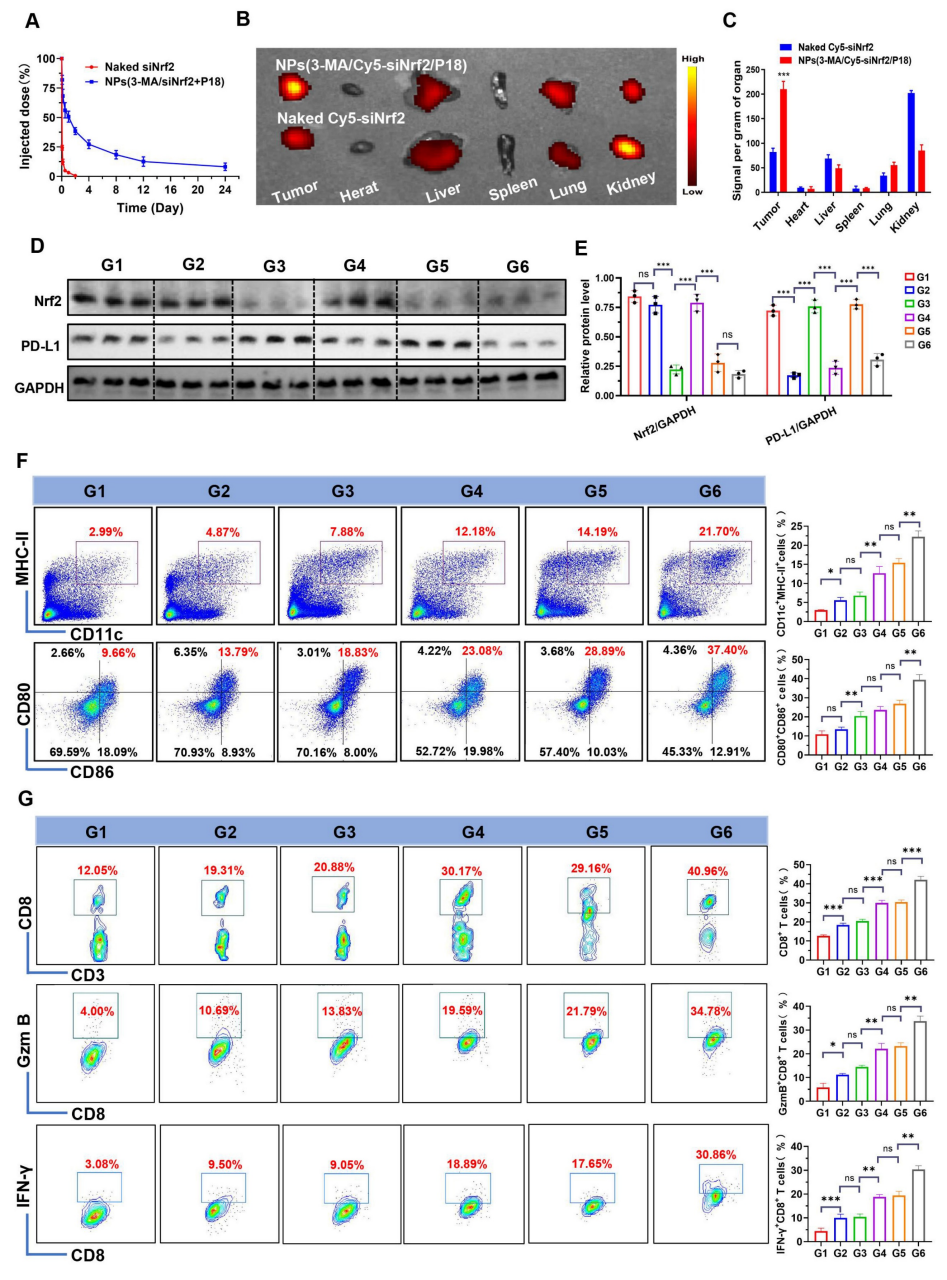
*In vivo* biodistribution, gene silencing, and immune activation induced by NPs(3-MA/Cy5-siNrf2/P-18) in 4T1 orthotopic tumor-bearing mice. (A) Blood circulation profiles of naked Cy5-siNrf2 and NPs(3-MA/Cy5-siNrf2/P-18) following intravenous administration in healthy mice, indicating enhanced stability and circulation time of the nanoparticle formulation. (B) *In vivo* fluorescence imaging of 4T1 orthotopic tumor-bearing mice at 24 h post-injection, showing tumor-targeted accumulation of NPs(3-MA/Cy5-siNrf2/P-18). (C) *Ex vivo* fluorescence images of major organs and tumors harvested from the mice in (B), confirming preferential tumor accumulation and biodistribution. (D, E) Western blot analysis and corresponding quantification of Nrf2 and PD-L1 protein expression in tumor tissues after treatment with the indicated formulations, demonstrating gene silencing and immune checkpoint regulation. (F) Flow cytometry analysis of matured DCs (CD11c⁺ CD80⁺CD86⁺) within the tumor microenvironment, indicating enhanced antigen-presenting activity. (G) Flow cytometry quantification of tumor-infiltrating CD8⁺ T cells, Granzyme B⁺ CD8⁺ T cells, and IFN-γ⁺ CD8⁺ T cells, assessing cytotoxic T cell activation in response to treatment. Treatment groups: G1: PBS; G2: NPs(3-MA/siCTL/P-18); G3: NPs(G0-C14/siNrf2/P-18); G4: NPs(3-MA/siCTL/P-18) + US; G5: NPs(G0-C14/siNrf2/P-18) + US; G6: NPs(3-MA/siNrf2/P-18) + US. Data are presented as mean ± SD (n = 5). Error bars represent standard deviation. Statistical analysis was performed using one-way ANOVA for multiple comparisons. Significance levels are indicated as *P < 0.05, **P < 0.01 and ***P < 0.001, ns, no significance.

Following the validation of NPs(3-MA/siNrf2/P-18)-mediated SDT in silencing Nrf2, downregulating PD-L1 expression, inhibiting protective mitophagy, and inducing intensive ICD to activate DCs, the *in vivo* antitumor efficacy was next evaluated. To determine whether these characteristics could enhance the anti-tumor immune response, NPs(3-MA/siNrf2/P-18) were intravenously administered into 4T1 orthotopic tumor-bearing mice. Due to the protective outer PEG chains 22, NPs(3-MA/siNrf2/P-18) exhibited prolonged blood circulation (**Figure 5A**) and efficient tumor accumulation (**Figure 5B-5C and Figure S10, Supporting Information**). This biodistribution profile resulted in significant silencing of Nrf2 and downregulation of PD-L1 in tumor tissue (**Figure 5D-5E**). The *in vitro* stability observed earlier correlated with the favorable *in vivo* pharmacokinetics, as the PEG shell minimized opsonization and clearance by the reticuloendothelial system. The prolonged circulation time allowed for enhanced tumor accumulation via enhanced permeability and retention effect, yielding a 2.8-fold increase in tumor-targeted delivery compared to free siRNA. To investigate the impact on immune cell activation, CD45^+^ cells were isolated from tumor tissues following NPs(3-MA/siNrf2/P-18) administration under US irradiation. Flow cytometry analysis showed a ~10-fold increase in CD11c^+^ MHC-II^+^ DCs and a ~4-fold increase in CD80^+^ CD86^+^ DCs (**Figure 5F**), indicating enhanced DCs maturation and antigen presentation capacity within the TME. As a result, significantly higher levels of tumor-infiltrating CD8^+^ T cells, Granzyme B^+^ CD8^+^ T cells, and IFN-γ^+^ CD8^+^ T cells were detected in tumor tissues (**Figure 5G**), reflecting robust activation of the adaptive immune response. Furthermore, due to PD-l1 downregulation by the 3-MA-loaded nanoparticle, immune checkpoint blockade was achieved, alleviating T cell exhaustion and contributing to the elevated percentages of CD8^+^ Granzyme B^+^ and CD8^+^ IFN-γ^+^ T cells. The gating strategy used for flow cytometry analysis in this experiment is shown in **Figure S11** (**Supporting Information**).

**Figure 6:**
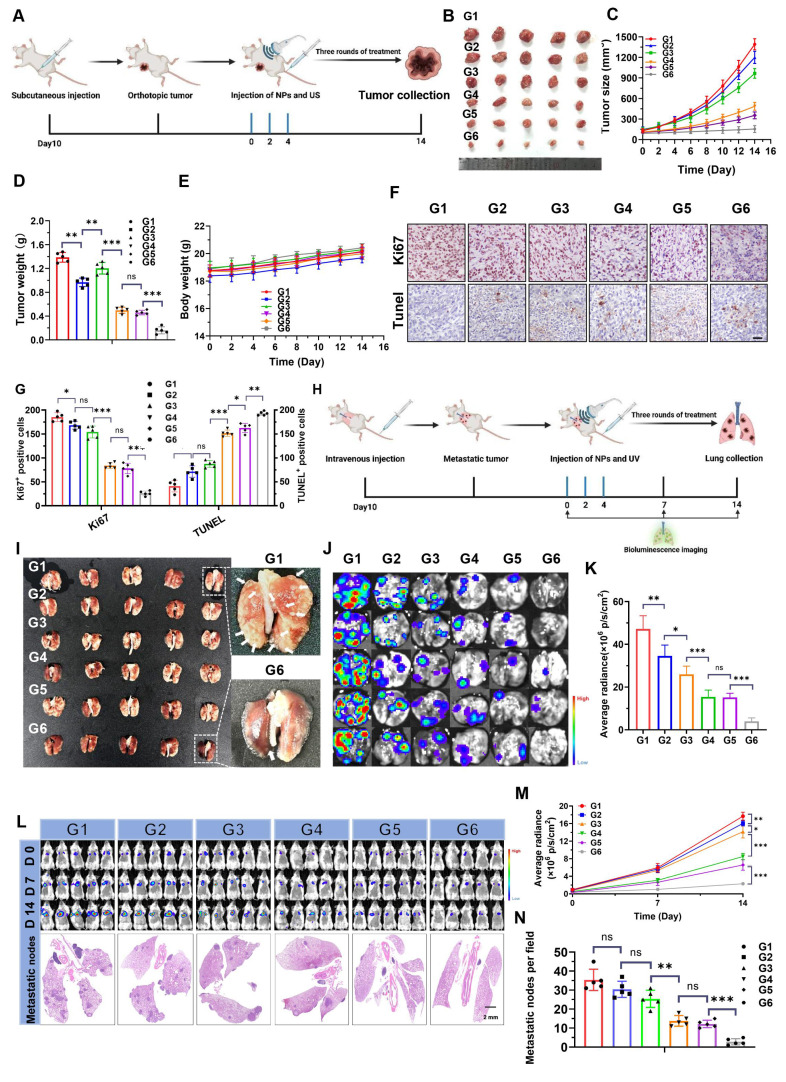
*In vivo* antitumor and antimetastatic efficacy of NPs(3-MA/siNrf2/P-18) under US irradiation. (A) Schematic illustration of the experimental timeline showing tumor inoculation, treatment schedule, and formulation details in 4T1 orthotopic tumor-bearing mice. Each treatment contained 5 mg/kg P-18, 6 mg/kg 3-MA, and/or 1 nM siNrf2 per mouse. (B) Representative images of excised primary tumors at the study endpoint. (C) Tumor growth curves for each treatment group. (D) Tumor weights measured at the endpoint, reflecting therapeutic efficacy. (E) Body weight changes of mice over the course of treatment, indicating systemic tolerability. (F) Representative IHC staining images of Ki67and TUNEL in tumor sections. (G) Quantification of Ki67⁺ and TUNEL⁺ cells per high-power field. (H) Schematic of the experimental setup for the lung metastasis model using luciferase-expressing 4T1 cells and treatment allocation. (I) Photographs of excised lungs showing visible metastatic nodules. (J) Representative bioluminescence imaging of lung metastases. (K) Quantitative analysis of lung bioluminescence intensity, indicating metastatic burden. (L) Whole-body bioluminescence imaging at days 0, 7, and 14 post-treatment, along with H&E staining of lung tissue sections to assess metastatic infiltration. (M) Quantification of whole-body bioluminescence signal intensity over time. (N) Quantification of metastatic nodules in H&E-stained lung sections. Treatment groups: G1: PBS; G2: NPs(3-MA/siCTL/P-18); G3: NPs(G0-C14/siNrf2/P-18); G4: NPs(3-MA/siCTL/P-18) + US; G5: NPs(G0-C14/siNrf2/P-18) + US; G6: NPs(3-MA/siNrf2/P-18) + US. Data are presented as mean ± SD (n = 5). Error bars represent standard deviation. Statistical analysis was performed using one-way ANOVA for multiple comparisons. Significance levels are indicated as *P < 0.05, **P < 0.01 and ***P < 0.001, ns, no significant,

Building on the encouraging results described above, the therapeutic efficacy of NPs(3-MA/siNrf2/P-18) *in vivo* was further evaluated in 4T1 orthotopic tumor-bearing mice under US irradiation (**Figure 6A**). As anticipated, intravenous administration of NPs(3-MA/siNrf2/P-18) combined with US irradiation significantly inhibited tumor growth without affecting overall body weight, indicating low systemic toxicity (**Figure 6B-6E**). Over the two-week treatment period, tumor volume in the PBS control group increased by approximately 13-fold, whereas tumor volume in the NPs(3-MA/siNrf2/P-18) + US group increased by only ~1.5-fold (**Figure S12, Supporting Information**). To dissect the individual contributions of mitophagy inhibition and Nrf2 silencing, NPs(3-MA/siCTL/P-18) and NPs(G0-C14/siNrf2/P-18) were administered with US irradiation. Each treatment led to ROS accumulation and moderate tumor growth inhibition over 14 days, underscoring the necessity of combinational therapy to achieve maximal therapeutic efficacy. This synergistic effect was further proven by histological analysis: TUNEL staining showed increased apoptosis, and Ki67 staining revealed reduced proliferation in tumor tissues treated with NPs(3-MA/siNrf2/P-18) (**Figure 6F-6G**). To evaluate the anti-metastatic potential of the NP, a luciferase-expressing 4T1 (Luc-4T1) lung metastasis model was established (**Figure 6H**) 8. Similar to the inhibition observed in orthotopic tumors, NPs(3-MA/siNrf2/P-18) treatment significantly suppressed lung metastases compared to all other treatment groups. This was evidenced by a lower number of metastatic nodules (**Figure 6I**), reduced bioluminescence signal from lung tissues (**Figure 6J-6K**), and diminished whole-body bioluminescence intensity (**Figure 6L, Figure 6M** and **Figure S13, Supporting Information**). Hematoxylin and eosin (H&E) staining further confirmed a marked reduction in metastatic nodules in lung sections (**Figures 6L** and **Figure 6N**). Notably, no apparent histological abnormalities were observed in major organs of mice treated with the NPs(3-MA/siNrf2/P-18) under US irradiation (**Figure S14, Supporting Information**), and no obvious fluctuations were detected in hematological parameters (**Figure S15, Supporting Information**) or in liver and kidney function tests (**Figure S16, Supporting Information**), confirming the favorable *in vivo* biosafety profile of NPs(3-MA/siNrf2/P-18) under US irradiation. In summary, the reduction-responsive RNAi NP demonstrated excellent anti-tumor and anti-metastatic efficacy *in vivo*, along with good biosafety and biocompatibility, supporting its potential for translational cancer therapy.

## Conclusion

This study presents an innovative approach that concurrently targets the mitophagy and Nrf2 pathways to enhance SDT by amplifying intracellular ROS generation within tumor cells. This strategy significantly curtails both progression and metastasis in breast cancer. The ROS-induced oxidative stress promotes apoptosis and triggers extensive ICD, leading to the release of DAMPs that promote DCs maturation and antigen presentation to CD8^+^ T cells. In addition, 3-MA downregulates PD-L1 expression via an NF-κB-dependent pathway, thereby mitigating T cell exhaustion and bolstering CD8^+^ T cell-mediated antitumor immunity. The reduction-responsive RNAi NP developed in this study proves to be a potent tool for augmenting therapeutic outcomes in breast cancer. Beyond SDT, the ROS amplification capability of this NP may also enhance other ROS-dependent modalities, including chemodynamic therapy, photodynamic therapy, radiotherapy, and chemotherapy. Furthermore, it synergizes effectively with immune checkpoint blockade. Collectively, the SDT-mediated, reduction-responsive RNAi NP represents a novel, versatile, and effective strategy for advancing multimodal cancer treatment.

## Supplementary Material

Supplementary figures and tables.

## Figures and Tables

**Table 1:** Primers for qRT-PCR

Gene	Forward	Reverse
Nfr2 (human)	CACATCCAGTCAGAAACCAGTGG	GGA ATGTCTGCGCCAAAAGCTG
Nfr2 (mouse)	CAGCATAGAGCAGGACATGGAG	GAACAGCGGTAGTATCAGCCAG
HO-1 (human)	CCAGGCAGAGAATGCTGAGTTC	AAGACTGGGCTCTCCTTGTTGC
HO-1 (mouse)	CACTCTGGAGATGACACCTGAG	GTGTTCCTCTGTCAGCATCACC
GR (human)	TATGTGAGCCGCCTGAATGCCA	CACTGACCTCTATTGTGGGCTTG
GR (mouse)	GTTTACCGCTCCACACATCCTG	GCTGAAAGAAGCCATCACTGGTG
NQO1 (human)	CCTGCCATTCTGAAAGGCTGGT	GTGGTGATGGAAAGCACTGCCT
NQO1 (mouse)	GCCGAACACAAGAAGCTGGAAG	GGCAAATCCTGCTACGAGCACT
SOD (human)	ACGCTGGCGAGGACGACCTG	GCTTCTTGCGCTCTGAGTGCTC
SOD (mouse)	GACCTGGTTGAGAAGATAGGCG	TGGCTGATGGTTGTACCCTGCA
GPX4 (human)	ACAAGAACGGCTGCGTGGTGAA	GCCACACACTTGTGGAGCTAGA
GPX4 (mouse)	CCTCTGCTGCAAGAGCCTCCC	CTTATCCAGGCAGACCATGTGC
