# Supplementary material for: Reduction-responsive RNAi nanoplatform for enhanced cancer sonoimmunotherapy via dual inhibition of mitophagy and Nrf2 pathways
Source: Theranostics. 2025 Jul 11;15(16):7973–89. doi: 10.7150/thno.112649 (PMC12374548; doi:10.7150/thno.112649)
Supplement: Supplementary file 1 — Supplementary figures and tables. [file thnov15p7973s1.pdf]

Supporting Information

of

Reduction-responsive RNAi nanoplatform for enhanced cancer sonoimmunotherapy via dual inhibition of mitophagy and Nrf2 pathways

Junyue Fang[a,b,c,d], Rui Xu[a,b,c], Yuan Cao[a,b,c], Zixuan Zhao[a,b,c], Weifan Li[a,b,c], Li Lin[a,b,c,e], Jingyi Hou[a,b,c,f], Xiaoding Xu[a,b,c], Phei Er Saw[a,b,c,g]**

[a] Guangdong Provincial Key Laboratory of Malignant Tumor Epigenetics and Gene Regulation, Medical Research Center, Sun Yat-Sen Memorial Hospital, Sun Yat-Sen University, Guangzhou 510120, P. R. China

[b] Guangzhou Key Laboratory of Medical Nanomaterials, Sun Yat-Sen Memorial Hospital, Sun Yat-Sen University, Guangzhou 510120, P. R. China

[c] Nanhai Translational Innovation Center of Precision Immunology, Sun Yat-Sen Memorial Hospital, Foshan, 528200, P. R. China

[d] Cellular and Molecular Diagnostics Center, Sun Yat-Sen Memorial Hospital, Sun Yat-Sen University, Guangzhou 510120, P. R. China

[e] Department of Dermatology, Sun Yat-Sen Memorial Hospital, Sun Yat-Sen University, Guangzhou 510120, P. R. China

[f] Department of Orthopedics and Department of Sports Medicine, Sun Yat-Sen Memorial Hospital, Sun Yat-Sen University, Guangzhou 510120, P. R. China

[g] Department of General Medicine, Sun Yat-Sen Memorial Hospital, Sun Yat-Sen University, Guangzhou 510120, P. R. China

*Correspondence, Email: xuxiaod5@mail.sysu.edu.cn; caipeie@mail.sysu.edu.cn

No.	NP1	NP2	NP3	NP4	NP5	NP6
N/P	19	38	57	76	95	114
3-MA (nmol)	160	320	480	640	800	960
3-MA EE%	75%	79%	84%	87%	93%	92%
siRNA (nmol)	1	1	1	1	1	1
siRNA EE%	65%	68%	71%	72%	76%	75%
P-18 (μmol)	180	180	180	180	180	180
P-18 EE%	45%	48%	51%	58%	62%	60%
Size (nm)	69	72	75	80	82	79
Zeta (mV)	-5.9	-5.3	-6.2	-7.5	-9.4	-8.6
PdI	0.15	0.19	0.21	0.15	0.2	0.25

Figure S1. Size, zeta potential, polydispersity index (PDI), siRNA encapsulation efficiency, 3-MA encapsulation efficiency and P-18 encapsulation efficiency of NPs(3-MA/siNrf2/P-18) prepared at different N/P molar ratios.

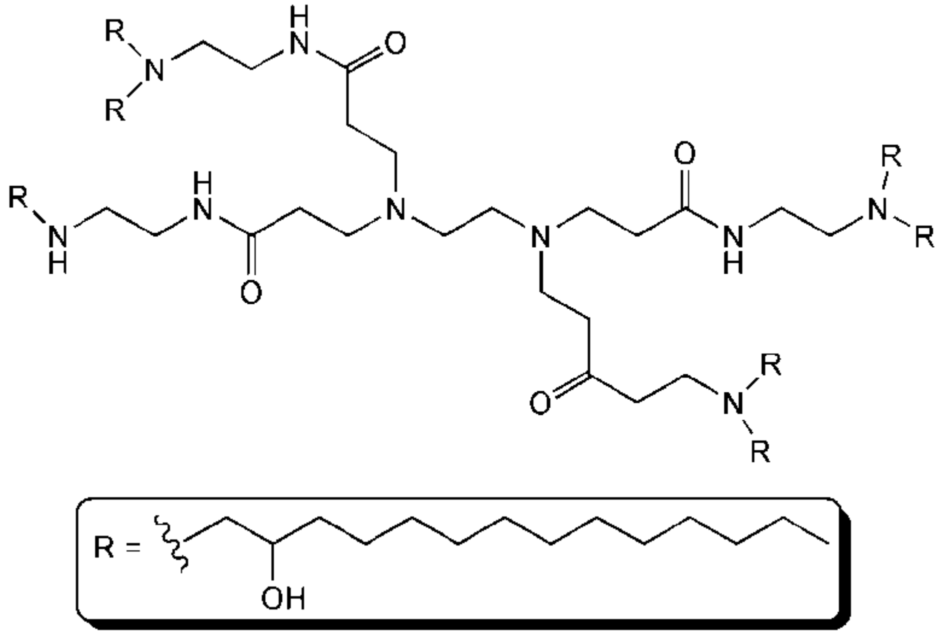

Figure S2. Chemical structure of amphiphilic cationic lipid-like compound G0-C14.

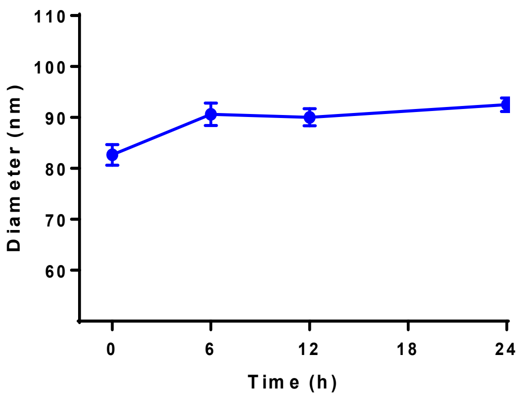

Figure S3. Size change of the NPs(3-MA/siNrf2/P-18) incubated in mouse plasma (1:1 v/v) at 37 °C for different time.

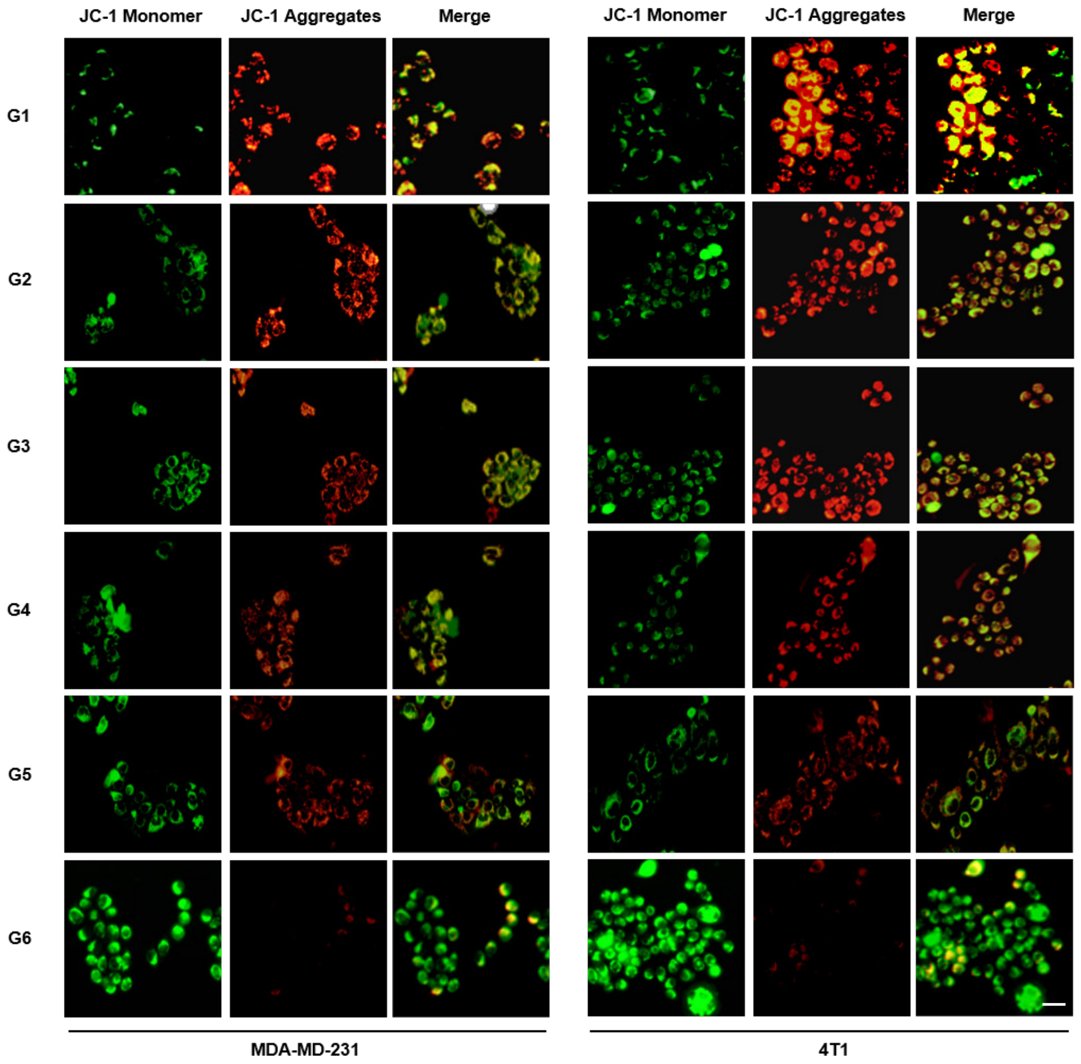

Figure S4. Confocal representative images of JC-1 staining for 4T1 and MDA-MD-231 cells under different treatments. (scale bar: 50 μm). G1: PBS; G2: NPs(3-MA/siCTL/P-18); G3: NPs(G0-C14/siNrf2/P-18); G4: NPs(3-MA/siCTL/P-18) + US; G5: NPs(G0-C14/siNrf2/P-18) + US; G6: NPs(3-MA/siNrf2/P-18) + US.

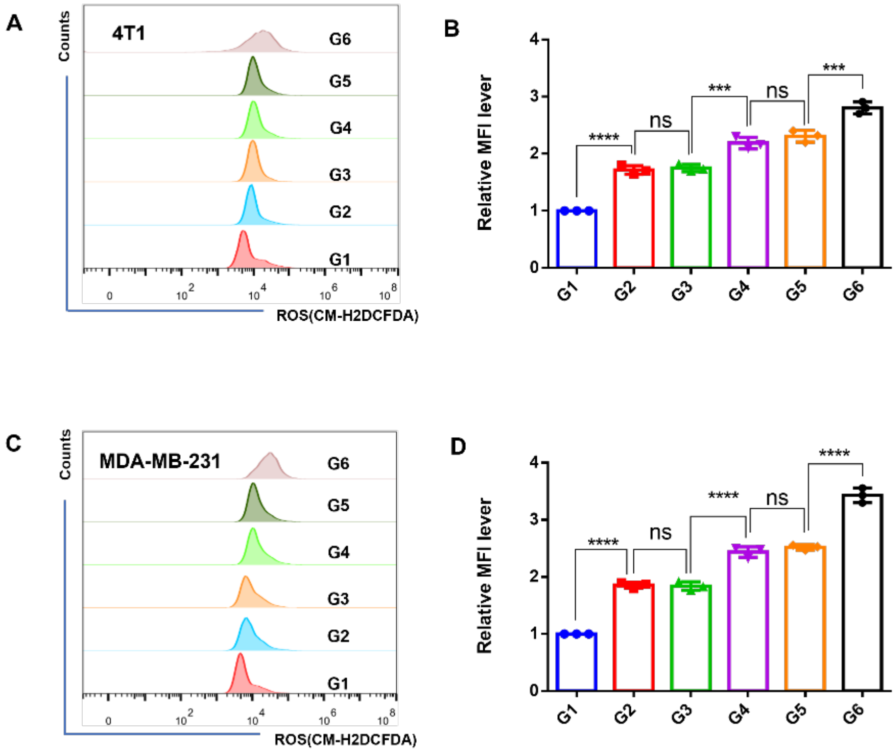

Figure S5. Using flow cytometry to detect MitoSOX Red staining for 4T1 and MDA-MB-231 cells under different treatments. G1: PBS; G2: NPs(3-MA/siCTL/P-18); G3: NPs(G0-C14/siNrf2/P-18); G4: NPs(3-MA/siCTL/P-18) + US; G5: NPs(G0-C14/siNrf2/P-18) + US; G6: NPs(3-MA/siNrf2/P-18) + US.

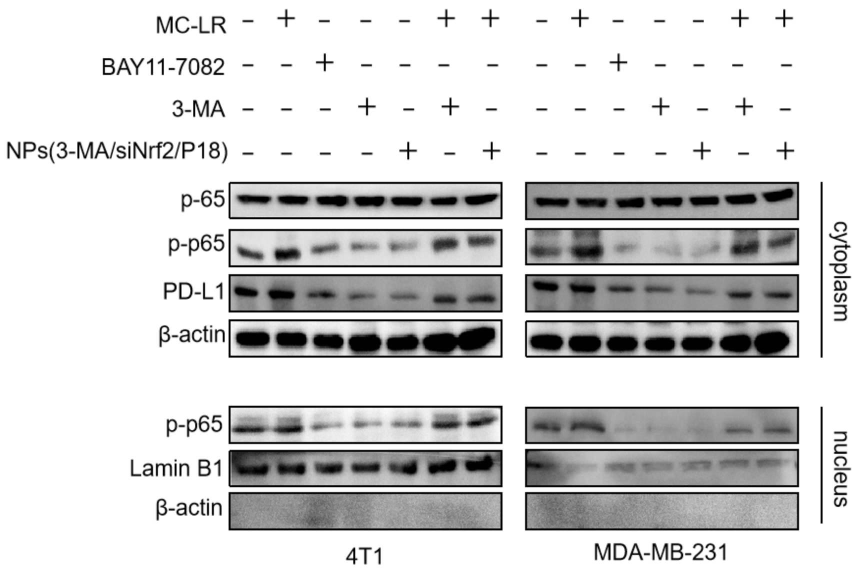

Figure S6. 3-MA and 3-MA-containing NP suppress PD-L1 expression via the NF-κB signaling pathway.

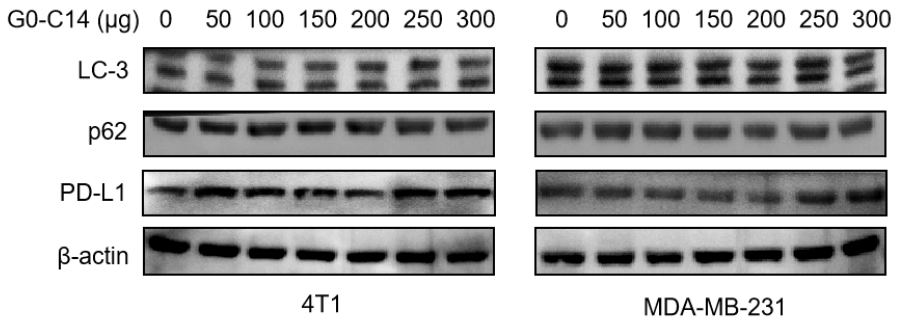

Figure S7. The effects of different concentrations of G0-C14 on autophagy or PD-L1 expression in 4T1 cells or 231 cells.

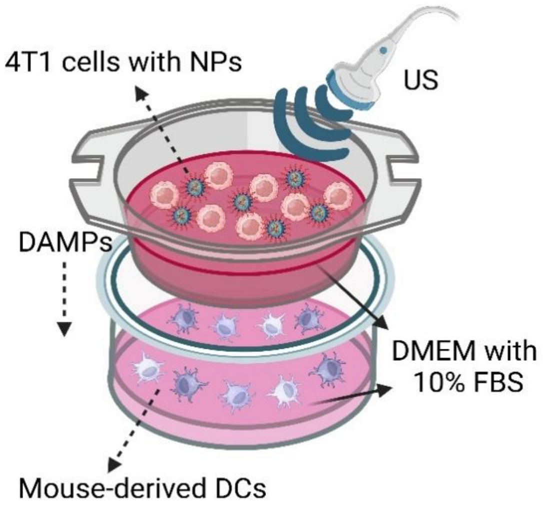

Figure S8. Schematic illustration of 4T1 cells co-cultured with mouse-derived DCs and the NPs(3-MA/siNrf2/P-18) in a Boyden chamber device with US irradiation.

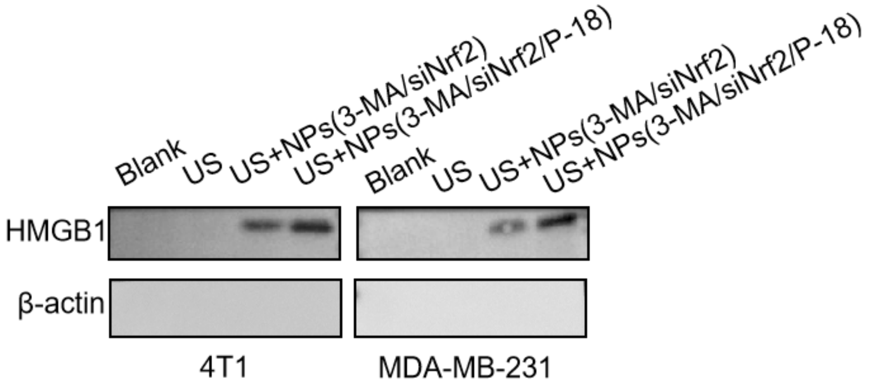

Figure S9. Evaluation of immunogenic cell death (ICD) in 4T1 and MDA-MB-231cells treated with ultrasound (US) alone.

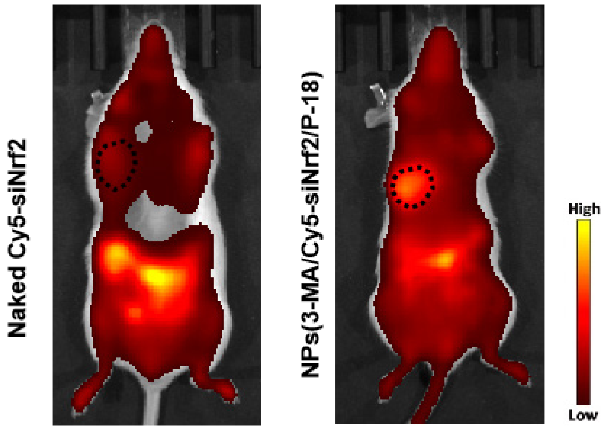

Figure S10. The biodistribution of naked Cy5-siNrf2 and NPs(3-MA/siNrf2/P-18) in situ tumor.

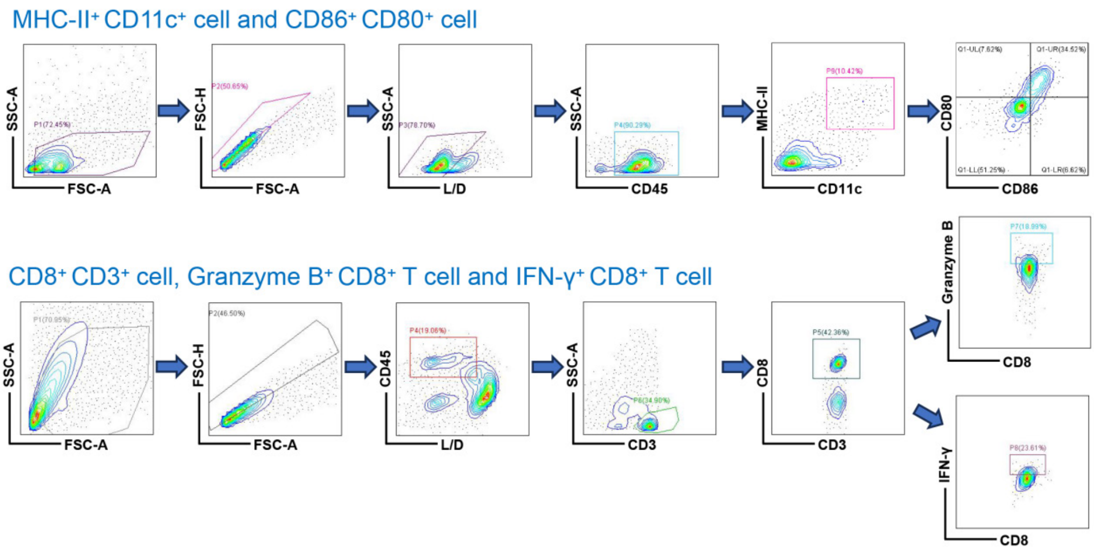

Figure S11. The gating strategy of flow cytometry analysis of DCs maturation and CD8$^+$ T cell activation.

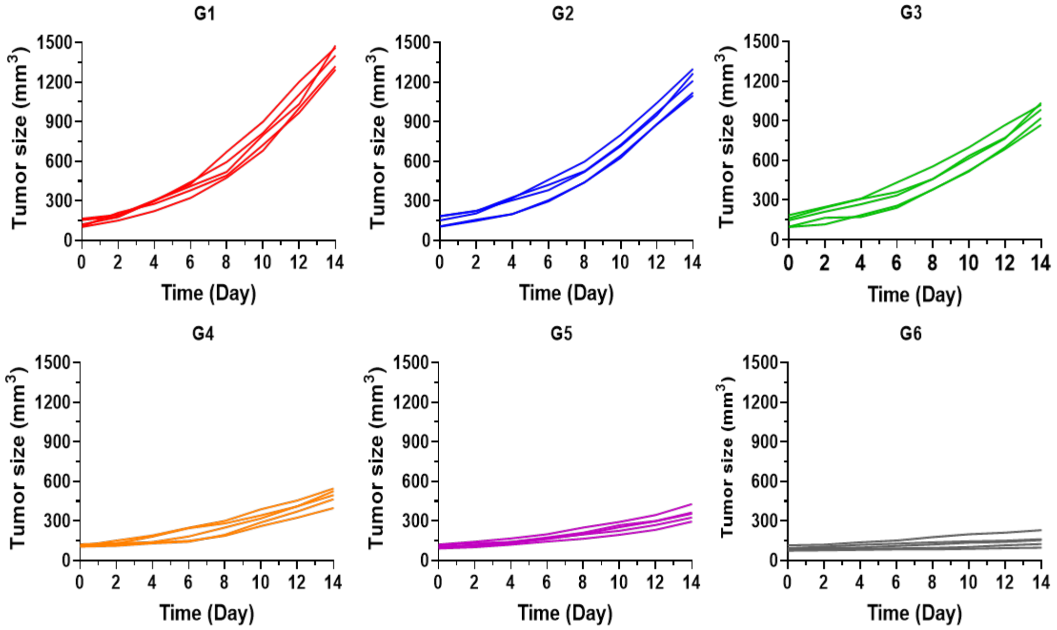

Figure S12. Tumor size of 4T1 orthotopic tumor-bearing mice treated with G1, G2, G3, G4, G5 and G6. G1: PBS; G2: NPs(3-MA/siCTL/P-18); G3: NPs(G0-C14/siNrf2/P-18); G4: NPs(3-MA/siCTL/P-18) + US; G5: NPs(G0-C14/siNrf2/P-18) + US; G6: NPs(3-MA/siNrf2/P-18) + US.

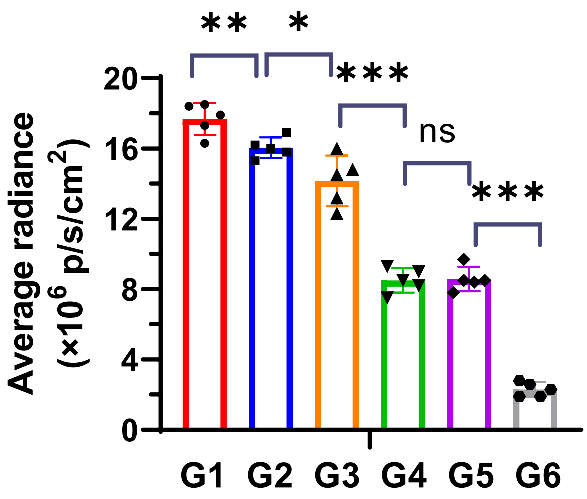

Figure S13. The average radiance of tumor burden at the endpoint (day 14) of each group. G1: PBS; G2: NPs(3-MA/siCTL/P-18); G3: NPs(G0-C14/siNrf2/P-18); G4: NPs(3-MA/siCTL/P-18) + US; G5: NPs(G0-C14/siNrf2/P-18) + US; G6: NPs(3-MA/siNrf2/P-18) + US. *ns*, no significance; * $p < 0.05$; ** $p < 0.01$; *** $p < 0.001$.

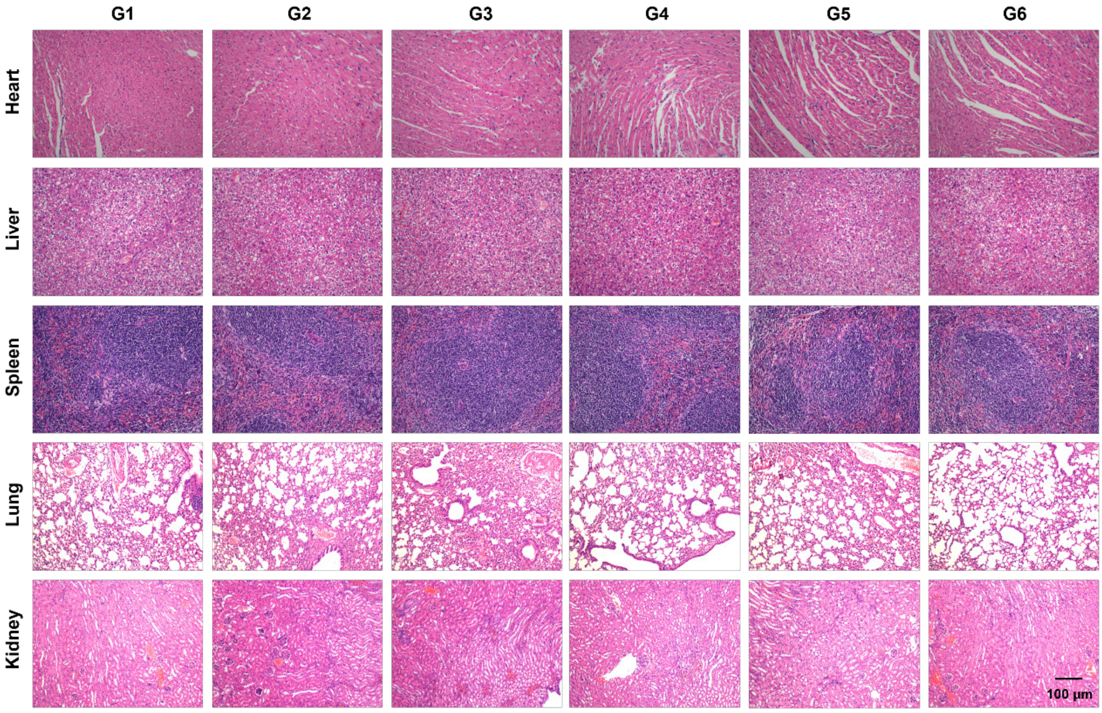

Figure S14. Histological analysis of the major organs of healthy mice received three consecutive intravenous injections of each group. (scale bar: 50 μm) G1: PBS; G2: NPs(3-MA/siCTL/P-18); G3: NPs(G0-C14/siNrf2/P-18); G4: NPs(3-MA/siCTL/P-18) + US; G5: NPs(G0-C14/siNrf2/P-18) + US; G6: NPs(3-MA/siNrf2/P-18) + US.

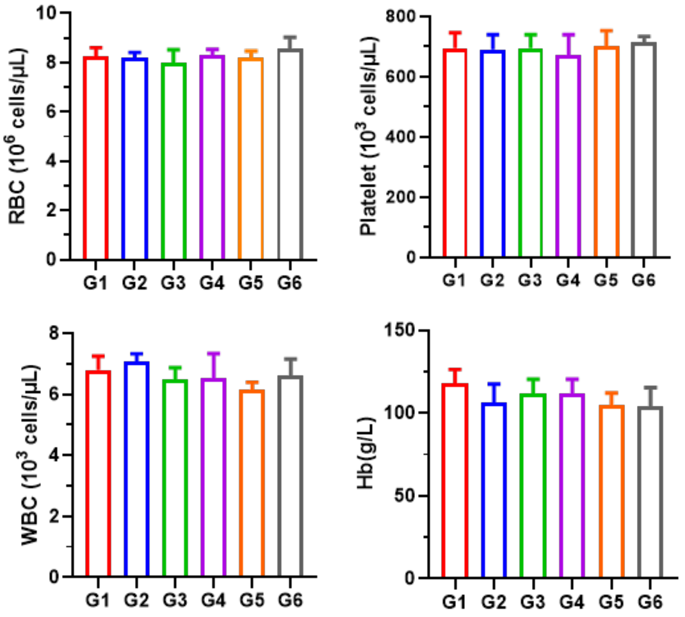

Figure S15. Number of red blood cell (RBC), white blood cell (WBC), and platelet, and the concentration of hemoglobin (Hb) in the blood of healthy mice received three consecutive intravenous injections of each group. G1: PBS; G2: NPs(3-MA/siCTL/P-18); G3: NPs(G0-C14/siNrf2/P-18); G4: NPs(3-MA/siCTL/P-18) + US; G5: NPs(G0-C14/siNrf2/P-18) + US; G6: NPs(3-MA/siNrf2/P-18) + US.

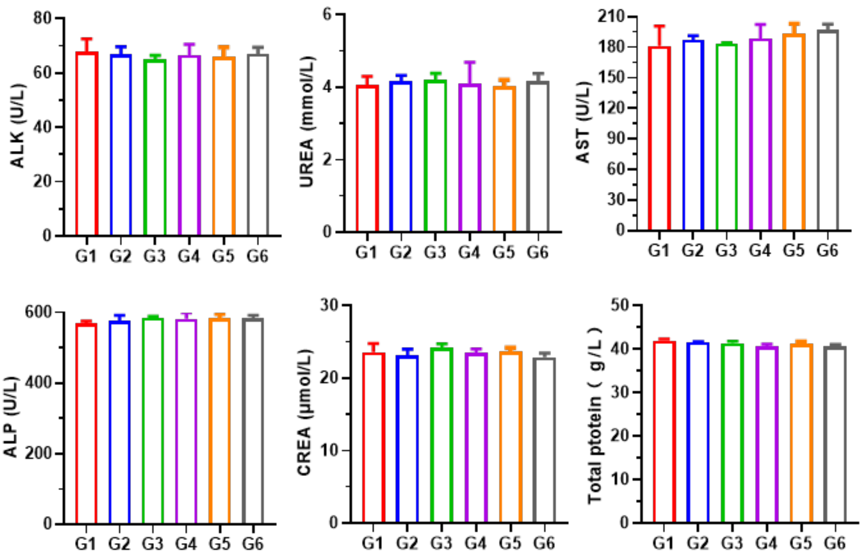

Figure S16. Serum levels of alanine aminotransferase (ALT), Urea, aspartate aminotransferase (AST), creatinine (CREA), total protein, and alkaline phosphatase (ALP) of healthy mice received three consecutive intravenous injections of each group. G1: PBS; G2: NPs(3-MA/siCTL/P-18); G3: NPs(G0-C14/siNrf2/P-18); G4: NPs(3-MA/siCTL/P-18) + US; G5: NPs(G0-C14/siNrf2/P-18) + US; G6: NPs(3-MA/siNrf2/P-18) + US.

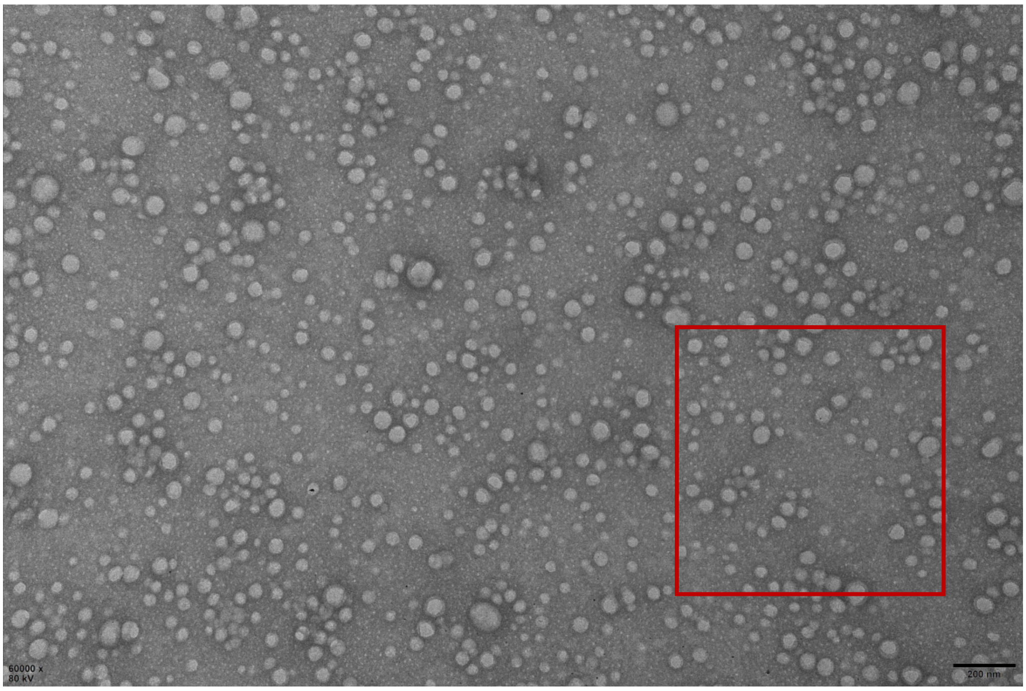

Figure R. Full-field TEM images of NPs(3-MA/siNrf2/P-18) (scale bar: 200 nm).
